# Antiradical Properties of N-Oxide Surfactants—Two in One

**DOI:** 10.3390/ijms22158040

**Published:** 2021-07-27

**Authors:** Agnieszka Lewińska, Julita Kulbacka, Marta Domżał-Kędzia, Maciej Witwicki

**Affiliations:** 1 Faculty of Chemistry, University of Wrocław, Joliot-Curie 14, 50-383 Wroclaw, Poland; 2 Department of Molecular and Cellular Biology, Faculty of Pharmacy, Wroclaw Medical University, Borowska 211A, 50-367 Wroclaw, Poland; julita.kulbacka@umed.wroc.pl; 3 Department of Biotransformation, Faculty of Biotechnology, University of Wroclaw, Joliot-Curie 14a, 50-383 Wroclaw, Poland; marta.domzal@uwr.edu.pl

**Keywords:** surfactants, antiradical properties, radical scavenging, antioxidant, EPR spectroscopy, UV-vis spectroscopy, DFT, DLPNO-CCSD(T)

## Abstract

Surfactants are molecules that lower surface or interfacial tension, and thus they are broadly used as detergents, wetting agents, emulsifiers, foaming agents, or dispersants. However, for modern applications, substances that can perform more than one function are desired. In this study we evaluated antioxidant properties of two homological series of N-oxide surfactants: monocephalic 3-(alkanoylamino)propyldimethylamine-N-oxides and dicephalic N,N-bis[3,3′-(dimethylamino)propyl]alkylamide di-N-oxides. Their antiradical properties were tested against stable radicals using electron paramagnetic resonance (EPR) and UV-vis spectroscopy. The experimental investigation was supported by theoretical density functional theory (DFT) and ab initio modeling of the X–H bonds dissociation enthalpies, ionization potentials, and Gibbs free energies for radical scavenging reactions. The evaluation was supplemented with a study of biological activity. We found that the mono- and di-N-oxides are capable of scavenging reactive radicals; however, the dicephalic surfactants are more efficient than their linear analogues.

## 1. Introduction

All living organisms are continuously exposed to a variety of harmful factors such as UV irradiation or environmental pollutants [1,2]. As an effect of these factors, the concentration of reactive radicals arises. To regain stability, the reactive radicals tend to undergo reduction, causing oxidative damage to biomolecules in the surroundings. Antioxidants prevent this process in biological systems and enable living cells to repair and renew. Providing natural antioxidants, e.g., from the group of curcuminoids or polyphenols, additionally with other antioxidant compounds, can significantly support patients during therapy in the case of various diseases, such as neurodegenerative or cancerous diseases [3,4,5,6]. The search for effective antioxidants has become one of the priorities of research in many scientific centers in the world because the efficacy of antioxidant compounds varies with structural factors and action mechanisms [7,8,9].

The external application of antioxidants plays an important role in pharmaceutical and cosmetic formulations. Modern cosmetic and pharmaceutical formulations are based on carriers of active substances in which the main role is played by compounds lowering the surface tension at the interface between two phases. Surfactants are amphipathic compounds with hydrophilic and hydrophobic fragments [10]. They are used as emulsifying substances in the production of creams and lotions. They also have foaming and wetting properties, which is why they are widely used in the production of detergents such as dishwashing liquid, soaps, or washing powders. Surfactants also increase the solubility of poorly soluble compounds and increase the penetration of active compounds, e.g., contained in skin cosmetics [11,12]. New applications of surfactants also include their use in carriers, e.g., in the form of nanoemulsions [13]. This approach allows not only for the delivery of active substances deep into a specific target site to increase the solubility of hydrophobic compounds but also for protecting them against the often harmful influence of external factors [14].

For modern applications, substances that can perform more than one function are desired. Hence, surfactants, which will not only reduce surface tension but will also have other properties, e.g., anti-microbial, antioxidant, or other, are sought. Non-ionic N-oxide surfactants exhibit very low toxicity and are biodegradable [15]. For these reasons, they are used in pharmaceuticals, cosmetics, and food products. In addition to their aggregation and emulsifying features that we reported previously [16], they also have antimicrobial and immunomodulatory properties [15], and they have been demonstrated to enhance antioxidant properties of (+)-usnic acid-loaded liposomes [15]. However, the antioxidant properties of N-oxide surfactants remain unexamined, and thus we attempted to characterize the antiradical properties of synthesized single head-single tail 3-(alkanoylamino)propyldimethylamine-N-oxides (C_n_PDA) and two head-single tail N,N-bis[3,3′-(dimethylamino)propyl]alkylamide di-N-oxides (C_n_(DAPANO)_2_) (structures are shown in Table 1).

To accomplish this aim, the radical scavenging activity of the N-oxides was evaluated using electron paramagnetic resonance (EPR) spectroscopy against two stable radicals: galvinoxyl (GO•) and 1,1-diphenyl-2-picrylhydrazyl (DPPH•). In the next step, the kinetics of the radical scavenging was monitored by UV-vis absorption spectroscopy. The EPR and UV-vis experiments were supplemented with the theoretical calculations at the density functional theory (DFT) and domain based local pair natural orbital coupled-cluster singles and doubles with perturbative inclusion of triples [DLPNO-CCSD(T)] theory levels. The biological examination of the N-oxide surfactants’ cytotoxicity and antioxidant properties on cell cultures of normal human cells was performed. The use of keratinocytes and fibroblasts allowed for the determination of surfactant interactions with skin and mucosa cells.

## 2. Results and Discussion

The studied bifunctional N,N-bis[3,3′-(dimethylamino)propyl]alkylamide di-N-oxide surfactants [C_n_(DAPANO)_2_; n = 10, 12, 14, 16], whose structure and properties are summarized in Table 1, were prepared in a straightforward procedure from readily available, inexpensive bio-reagents under mild conditions [18]. Other synthesized surfactants—C_n_PDA—were prepared using a procedure adapted from Piasecki et al. [19]. Hence, the synthetic procedure applied to obtain C_n_(DAPANO)_2_ and C_n_PDA regards an important principle of green chemistry.

**Figure 1:**
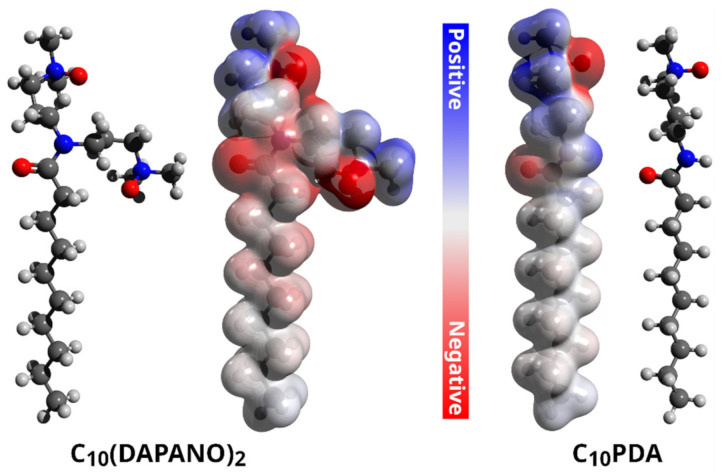
Electrostatic potential maps for the studied C_10_(DAPANO)_2_ and C_10_PDA surfactants, which were computed at the DFT B3LYP/def2-TZVP level with implicit CPCM solvation. Red surfaces enclose a negative charge, while positive zones show as blue surfaces.

Previously, we showed that the studied N-oxide surfactants have a profound tendency to form micelles in water, and according to our potentiometric titrations, they can be considered as nonionic at a pH close to 7, which is a physiological condition [17]. As shown by DFT calculations, such nonionic C_n_(DAPANO)_2_ and C_n_PDA surfactants feature locally negative and positive charge zones in the N-oxide heads and on the carbonyl group (Figure 1). The positive and negative charge is strongly localized on nitrogen and oxygen atoms, respectively. As discussed by Durán-Álvarez et al. [20], such a strong charge localization reflects a hard electrostatic nature of atoms being the charge carriers in C_n_(DAPANO)_2_ and C_n_PDA. There is, however, an important difference in charge distribution between C_n_(DAPANO)_2_ and C_n_PDA. As exemplified by C_10_(DAPANO)_2_, for the di-N-oxide surfactants, the negative charge diffuses onto the first five carbon atoms of the hydrophobic tail. This negative charge diffusion is not observed for the mono-N-oxides, and thus they should have increased lipophilicity in comparison with their dicephalic counterparts. The COSMO-RS calculated partition coefficients P in a biphasic system of n-octanol and water (given in Table 1 as logP) amply corroborate this conclusion as the logP values for the homologs of the C_n_(DAPANO)_2_ series are significantly smaller than for C_n_PDA.

### 2.1. Radical Scavenging by EPR

A reliable approach to evaluate radical scavenging activity (or antiradical properties) is using a stable radical, whose concentration gradually decreases because of the reduction by an antiradical compound. This approach is based on the logical assumption that if a compound can react with the stable radical, it is certainly capable of reacting with much more aggressive radicals, such as •OH. The amount of stable radical reduced/remaining in the sample depends on the power (kind) of the antiradical compound (antioxidant) [7,21].

**Figure 2:**
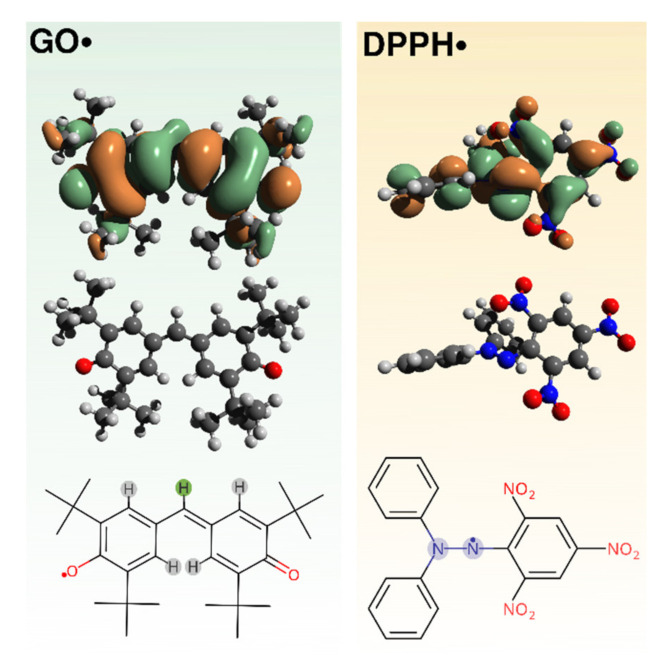
Structure of 2,2-diphenyl-1-picrylhydrazyl (DPPH•) and galvinoxyl (GO•) radical along with SOMOs isosurfaces (cutoff isovalue 0.02 e/bohr^−3^) calculated at the B3LYP/def2-TZVP theory level. Atoms giving rise to the hyperfine splitting observed in the EPR spectra are highlighted.

To test the scavenging activity of C_n_(DAPANO)_2_ and C_n_PDA, we used galvinoxyl (GO•) and 2,2-diphenyl-1-picrylhydrazyl (DPPH•) radicals. Their structures, along with singly occupied molecular orbitals (SOMOs), are shown in Figure 2. The SOMOs show that the unpaired electron in DPPH• and GO• is highly delocalized and this delocalization increases the stability of these two radicals. The electronic structures of GO• and DPPH• were previously discussed in detail [8,9,22,23,24].

We began with an attempt to determine if the N-oxide surfactants can participate in the radical scavenging process when micelles are well-formed. Thus, the reaction mixtures containing a surfactant at C/CMC = 100, which is a typical concentration found in formulations, and GO• or DPPH• at 2 mM were prepared along with the control samples and monitored over time (solutions of DPPH• and GO• without surfactants).

**Figure 3:**
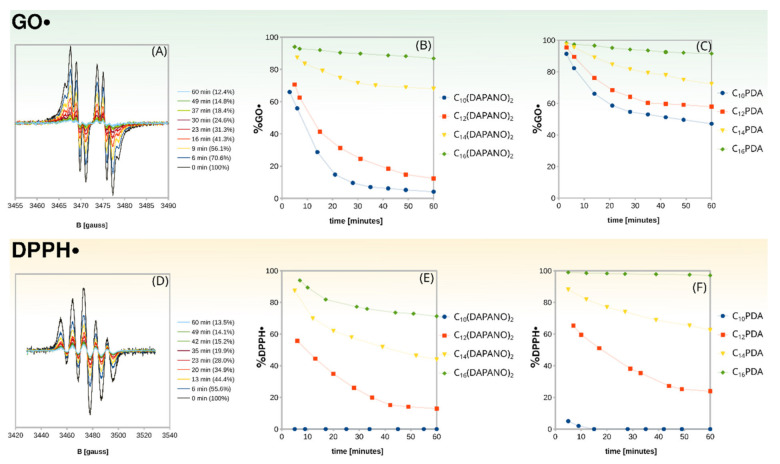
The scavenging activity of C_n_(DAPANO)_2_ and C_n_PDA monitored with EPR spectroscopy: changes in the GO• absorption due to its reaction with C_12_(DAPANO)_2_ (**A**); changes in the GO• concentration due to its reaction with C_n_(DAPANO)_2_ and C_n_PDA [(**B**) and (**C**), respectively]; changes in the DPPH• absorption due to its reaction with C_12_(DAPANO)_2_ (**D**); changes in the DPPH• concentration due to its reaction with C_n_(DAPANO)_2_ and C_n_PDA [(**E**) and (**F**), respectively].

As the antiradical activity of the N-oxide surfactants could have been low, the decay of GO• and DPPH• in the presence of the di- and mono-N-oxide surfactants was monitored with electron paramagnetic resonance (EPR) spectroscopy, which is an extremely sensitive technique for the radical systems [25,26,27]. DPPH• and GO• have well-defined EPR spectra, and a clear decrease in their intensity was observed for the N-oxide surfactants as exemplified by C_12_(DAPANO)_2_ in Figure 3. An intensity decline was not observed for the control samples. Hence, the EPR experiments revealed the antiradical activity of C_n_(DAPANO)_2_ and C_n_PDA surfactants.

The scavenging capacity of the surfactants was judged quantitatively by measuring the percentage of remaining GO• and DPPH• after a fixed reaction time. The relative concentration of GO• and DPPH• was calculated from Equation (1):

(1) $$remaining\;radical\left(\%\right)=\frac{I_{t}}{I_{0}}\times 100\%$$

where $I_{0}$ and $I_{t}$ correspond to the EPR signal integral intensity of a radical in the absence and presence of the N-oxide surfactants after a fixed time, respectively.

These results are given as plots in Figure 3. The concentration of DPPH• and GO• underwent a decrease for both homologous series, but the reduction of radicals was less efficient with the increasing length of the surfactant alkyl chain. What stands out in Figure 3 is that the dicephalic surfactants scavenged DPPH• and GO• significantly faster than their linear counterparts. This is evident in the case of C_12_(DAPANO)_2_ and C_12_PDA. After 60 min of reaction, the former reduced the number of GO• by 88% and DPPH• by 86%, while the latter by 59% and 24%, respectively. From Figure 3, we can see that both di- and mono-N-oxides scavenge DPPH• more actively, and therefore DPPH• was used in our further studies as it seems to better mimic more reactive radicals such as •OH. Moreover, DPPH• is reliably used in similar studies [8,21,28].

### 2.2. Radical Scavenging by UV-Vis

The EPR studies for C/CMC = 100 imply that the rate of radical scavenging is higher for C_n_(DAPANO)_2_ than for their linear analogues. To verify this, the reaction of DPPH• with each di-N-oxide surfactant was monitored using pseudo-first-order kinetics. In this part of our work, we used UV-vis absorption spectroscopy since such experiments are quicker to set up in comparison with EPR, which requires careful tuning of the spectrometer after the sample is placed in the resonator, and thus the beginning of the reaction is difficult to monitor with EPR.

**Figure 4:**
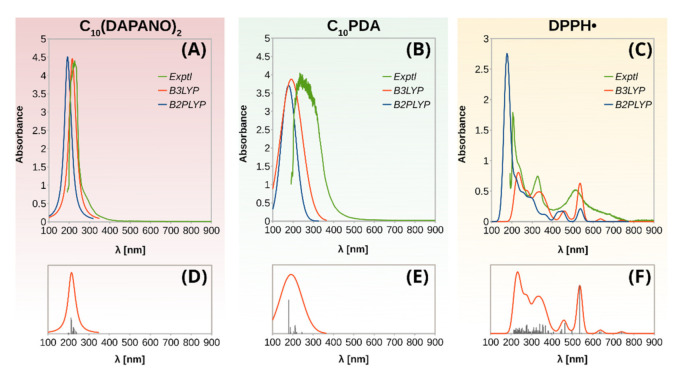
Experimental and theoretically computed UV-vis absorption spectra of C_10_(DAPANO)_2_ (**A**), C_10_PDA (**B**), and DPPH• (**C**). The predicted spectra were simulated by associating a single Gaussian (for C_10_PDA and DPPH•) or Lorentzian [for C_10_(DAPANO)_2_] to each computed transition. The transitions computed at the B3LYP theory level are shown in (**D**–**F**) for C_10_(DAPANO)_2_, C_10_PDA, and DPPH•, respectively.

**Figure 5:**
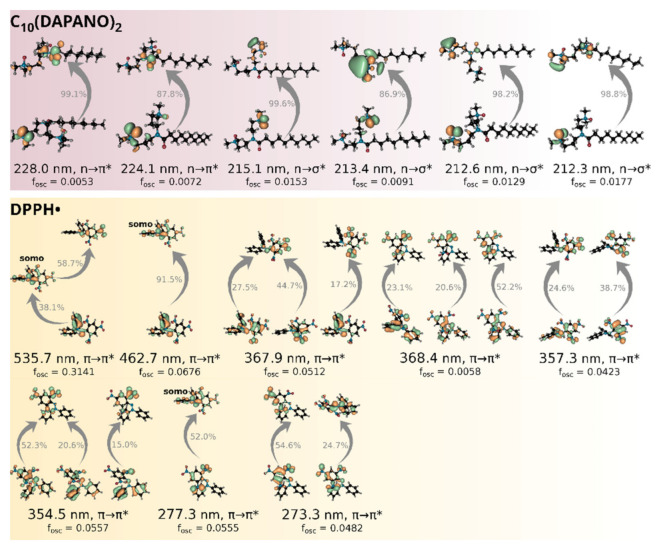
Natural transition orbitals from the time-dependent B3LYP/def2-TZVP computations picturing the most important electronic excited states for C_10_(DAPANO)_2_ and DPPH•. For each excited state, only the transitions contributing more than 10% are shown.

In contrast to EPR spectroscopy, however, in which only signals due to DPPH• and GO• were observed, to evaluate antiradical activity by UV-vis measurements meaningfully, the used stable radical is required to have absorption maxima outside the surfactant’s absorption range. As demonstrated by Figure 4, this is the case of the DPPH• selected to investigate the antiradical properties of the C_n_(DAPANO)_2_ and C_n_PDA surfactants series. To fully understand the absorption of DPPH• and the surfactants in the UV-vis range, the time-dependent density-functional theory (TD-DFT) calculations were carried out. Their results are summarized in Figure 4 and Figure 5. The use of the B3LYP functional led to a highly satisfactory agreement between the theoretical and experimental spectra, and henceforth the results of the TD-DFT B3LYP/def2-TZVP calculations are given in the text and Figure 5.

As exemplified by C_10_(DAPANO)_2_ and C_10_PDA, the di- and mono-N-oxide surfactants show strong absorption at the range of 190–420 nm with a maximum at approximately 205 nm (Figure 4). As illustrated by the natural transition orbitals (NTOs) [29] from TD-DFT computations, the heads of the surfactants play the role of chromophores. The observed absorption results from the excitation of the oxygen p-type lone pairs to the π* and σ* orbitals (Figure 5).

The presence of a delocalized electron in DPPH• gives rise to the deep violet color. The decrease in the DPPH• absorption at 517 nm is associated with the undergoing scavenging of this radical by an antioxidant. As Figure 4 demonstrates, this absorption maximum is clearly outside the C_n_(DAPANO)_2_ and C_n_PDA absorption range, and thus it can be used to evaluate radical scavenging properties of the N-oxide surfactants. The energy of the electronic transition responsible for this absorption band was underestimated by the time-dependent B3LYP computations by less than 0.1 eV (expt: 2.40 eV; cald: 2.31 eV), which is a highly satisfactory agreement [30,31]. The analysis of NTOs reveals that two individual pairs of molecular orbitals contribute strongly to this excitation. The first pair with the contribution of 38.1% is the transition of an electron from a π-type doubly occupied molecular orbital to the SOMO, and the second pair defines the dominant transition from the SOMO to a π-type unoccupied orbital (contribution of 58.7%).

Although not well resolved in the experimental spectrum, another absorption band is present for DPPH• at approximately 463 nm and dominated by the promotion of the β-type electron to the SOMO. From about 400 nm towards shorter wavelengths, the excited states are increasingly numerous, and a detailed characterization of these among those which have more significant intensity is given in Figure 5.

**Figure 6:**
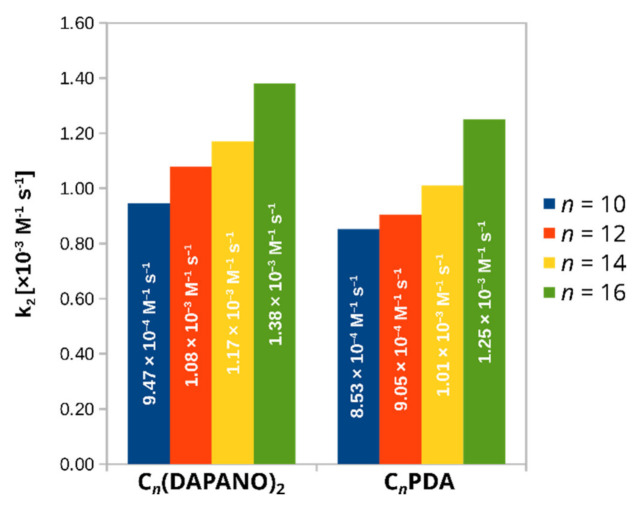
Comparison of the second-order rate constants k_2_ for the reaction between the N-oxide surfactants and DPPH•.

After confirming that the absorption maximum at 517 nm of DPPH• is outside the surfactant’s absorption range, the rate of the DPPH•-scavenging reaction of the N-oxides was measured by monitoring the decrease in absorbance of this maximum. To ensure that the decay of DPPH• obeyed pseudo-first-order kinetics, the concentration of surfactants was at least a 54-fold excess of the DPPH• concentration. As explained in SI, the calculated pseudo-first-order rate constants, k_obs_, increased linearly with the concentration of C_n_(DAPANO)_2_ and C_n_PDA (shown in SI). The second-order rate constants k_2_ for the reaction between the N-oxides and DPPH• were determined from the slopes of the linear plots of k_obs_ vs. the concentration of the surfactants and are given in Figure 6.

In comparison with C_n_PDA, the values of k_2_ for the dicephalic homologs were 11% 19%, 16%, and 10% higher for C_10_(DAPANO)_2_^,^ C_12_(DAPANO)_2_, C_14_(DAPANO)_2,_ and C_16_(DAPANO)_2_, respectively. The observed difference between the values of k_2_ strongly suggests that the chemical structure of C_n_(DAPANO)_2_ and C_n_PDA has an important effect on radical scavenging. This was explored using computational techniques and is discussed below.

The changes in the values of k_2_ demonstrate a faster reduction of DPPH• by the di-N-oxide surfactants, and this finding stays in line with our EPR measurements (Figure 3). In contrast to the EPR findings, however, the determined values of k_2_ show that the ability of the N-oxide surfactants to scavenge radicals increases with the length of the aliphatic chain. In the EPR tests of antiradical activity, the C/CMC = 100 conditions were applied. This allowed us to test this property at concentration characteristic of cosmetic formulations and for the surfactant systems with the same state of the formation of the micelles. However, there are drawbacks associated with the use of C/CMC = 100: the molar concentrations of the surfactants are higher for the surfactants with a larger CMC, that is, the ones with shorter alkyl chains. In the EPR measurements, we thus observed that the increased concentrations prevailed upon the greater antiradical activity of the surfactants with a lengthening hydrocarbon chain. The mentioned drawback of the EPR tests was overcoming the kinetic UV-vis experiments conducted under the pseudo-first-order condition.

### 2.3. Theoretical Calculations

To obtain a deeper insight into the antiradical properties, the thermodynamics of these processes for C_10_(DAPANO)_2_ and C_10_PDA was evaluated using the DFT approach (B3LYP, B2PLYP, and M06-2X) and the ab initio coupled cluster-type method [DLPNO-CCSD(T)]). The DLPNO-CCSD(T) method has been reported to reproduce results of the “golden standard” canonical CCSD(T) while remaining computationally feasible for real chemical systems [32,33,34,35,36,37]. Moreover, the excellent performance of the DLPNO-CCSD(T) method has been confirmed for the determination of the energetics of the hydrogen atom transfer reaction [38].

Two different mechanisms were considered. One of them, shown in Equation (2), assumes that a radical (*R•*) removes a hydrogen atom from the surfactant (*surf−H*) that itself transforms into a radical (H atom transfer, HAT):

*R• + surf−H → R−H + surf•* (2)

The one-electron transfer (Equation (3)) was the second mechanism under theoretical investigation. In this mechanism the surfactant was assumed to contribute an electron to a radical, becoming itself a radical cation in the process:

*R• + surf−H → R^−^ + surf−H^•+^* (3)

The two mechanisms were previously considered in the context of the antiradical activity of phenolic compounds and radical activity of insecticides in the soil environment [32,39,40,41,42,43,44].

In the hydrogen atom transfer, the X−H bond dissociation enthalpy (BDE) should be considered a crucial parameter [32,39,40,43], as the more weakly bonded H atoms would be more active in the scavenging reaction given in equation 2. BDE was calculated at 298 K as the enthalpy difference for the reaction [40,41,43,45]:

*surf*−*H *→* surf*• + •*H* (4)

**Figure 7:**
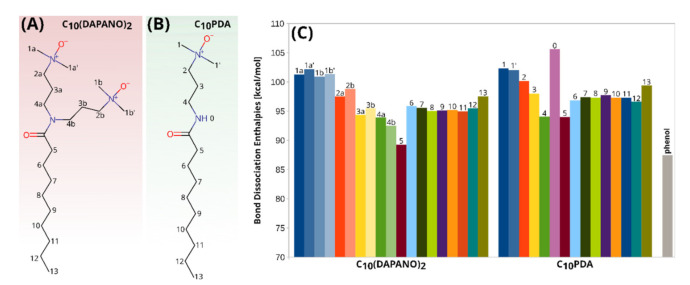
Labeling of atoms forming bonds with hydrogens in C_10_(DAPANO)_2_ and C_10_PDA molecule in (**A**,**B**), respectively, along with dissociation enthalpies calculated for these bonds at the DLPNO-CCSD(T)/ cc-pVTZ level at 298 K (**C**). The results of the B3LYP/def2-TZVP, B2PLYP/def2-TZVP and M06-2X/def2-TZVP calculations are given in Table S1. For comparison, BDE for phenol is shown.

Before proceeding with a detailed analysis, it is reasonable to compare the performance of the computational models. Table S1 in Supplementary Materials shows that all the employed methods gave a consistent outcome. The maximum difference between the values of BDE calculated with various methods was found to be 4%, and thus the conclusion is expected to be method-independent. In the further discussion of BDE, the values from the DLPNO-CCSD(T) calculations are used. For the sake of discussion clarity, all atoms forming bonds with hydrogen were labeled as shown in Figure 7, which also shows the values of BDE for these bonds predicted at the DLPNO-CCSD(T) theory level.

The highest value of BDE (105.6 kcal/mol) was found for the N−H bond in the C_10_PDA molecule, and thus this hydrogen atom should be the least active in the HAT mechanism. For this mono-N-oxide surfactant, the BDE values predicted for the hydrogen atoms bonded to the carbon atoms are in the range of 90.0–102.3 kcal/mol. The values from the upper limit of this range were predicted for the terminal carbon atom of the hydrophobic tail (C13), and carbon atoms bonded to the nitrogen atoms of the N-oxide moieties (C1 and C1’). The lowest BDE was found for the C4−H and C5−H bonds, that is 94.1 and 94.0 kcal/mol, respectively, showing that these hydrogen atoms should be the most active in the H-abstraction mechanism in the case of the mono-N-oxide surfactants.

C_10_(DAPANO)_2_ differs in structure from and C_10_PDA by an additional 3,3′-(dimethylamino)propyl substituent at the nitrogen atom of the amide group. Based on our EPR and UV-vis measurements, this structural alteration is expected to bring about dissimilar reactivity and it manifests in the values of BDE. As shown in Figure 7, the values of BDE calculated for the terminal carbon atom of the hydrophobic tail (C13) and carbon atoms bonded to the nitrogen atoms of the N-oxide group (C1a, C1a’, C1b and C1b’) very closely resemble their C_10_PDA counterparts. These values for the C4a−H and C4b−H bond are 93.9 and 92.5 kcal/mol, respectively, and do not differ from the C4−H bond dissociation enthalpy for C_10_PDA. However, the homolytic breaking of C5−H in C_10_(DAPANO)_2_ requires only 89.3 kcal/mol. This is significantly less than the energy necessary to enforce this process for the C5−H bond in the C_10_PDA molecule (94.0 kcal/mol) and correlates with the higher rates of reactions between the di-N-oxide surfactants and DPPH•. Moreover, according to our DLPNO-CCSD(T) calculations, the analogical process for O−H in phenol requires 87.8 kcal/mol, which is comparable to the BDE for C5−H in C_10_(DAPANO)_2_.

To compare the capacity of the mono- and di-N-oxide surfactants to donate an electron to a radical (one-electron transfer), ionization potentials (IP) were computed for C_10_PDA and C_10_(DAPANO)_2_ as the enthalpy difference between the surfactants and their radical cations (surf−H^•+^). This approach has been proven successful in the case of phenolic compounds and carbamates [40,41,43,44]. All the calculated values of IP are listed in Table S2 in Supplementary Materials. It is important to note that the ones obtained at the DLPNO-CCSD(T) theory level for C_10_PDA and C_10_(DAPANO)_2_ are up to 6 kcal/mol higher in comparison with the results of DFT methods. Considering that the DFT performance in the IP calculations can be unsatisfactory [46,47], the values obtained with the ab initio DLPNO-CCSD(T) method should be considered more accurate. They show that the IP of C_10_(DAPANO)_2_ is only about 1.1 kcal/mol lower than that of C_10_PDA (147.8 and 148.9 kcal/mol, respectively).

Computational techniques were also used to compare the energetic effect of the two mechanisms. The Gibbs free energies (ΔG) were calculated for the reactions given in Equation (2) (H atom abstraction) and Equation (3) (one-electron transfer) at 298 K. Two different radical substrates were considered, DPPH• and •OH. The results are summarized in Table S3 in Supplementary Materials.

Regardless of the radical substrate and the used method in the one-electron transfer, the ΔG values are positive. The DLPNO-CCSD(T) method shows that ΔG for the one-electron transfer between the two surfactants and the two radicals is moderately high. To illustrate, for C_10_(DAPANO)_2_, ΔG is 45.6 and 46.6 kcal/mol for the electron transfer to •OH and DPPH•, respectively. For C_10_PDA these values become slightly higher, that is, 47.7 and 48.7 kcal/mol, respectively.

In contrast to one-electron transfer, the ΔG values predicted for H atom abstraction are noticeably less positive for DPPH• and clearly negative for •OH. For example, the ΔG predicted for C_10_(DAPANO)_2_ at the DLPNO-CCSD(T) level is −29.2 and 11.8 kcal/mol for •OH and DPPH•, respectively, and for C_10_PDA, −19.9 and 21.1 kcal/mol, respectively. Although in the case of DPPH the reaction is not spontaneous, it was proven by the UV−vis spectroscopy experiments that the input of energy at 298 K suffices to sustain the radical scavenging process. It is also notable that the predicted ΔG values for the H atom abstraction closely correlate with different activity of the N-oxide surfactants. In comparison with C_10_PDA, the ΔG values predicted for more reactive C_10_(DAPANO)_2_ are noticeably less positive for DPPH• and more negative for •OH. All in all, our calculations distinctively suggest that for the N-oxide surfactants, the occurrence of the H atom transfer is thermodynamically more probable.

### 2.4. Biological Activity

**Figure 8:**
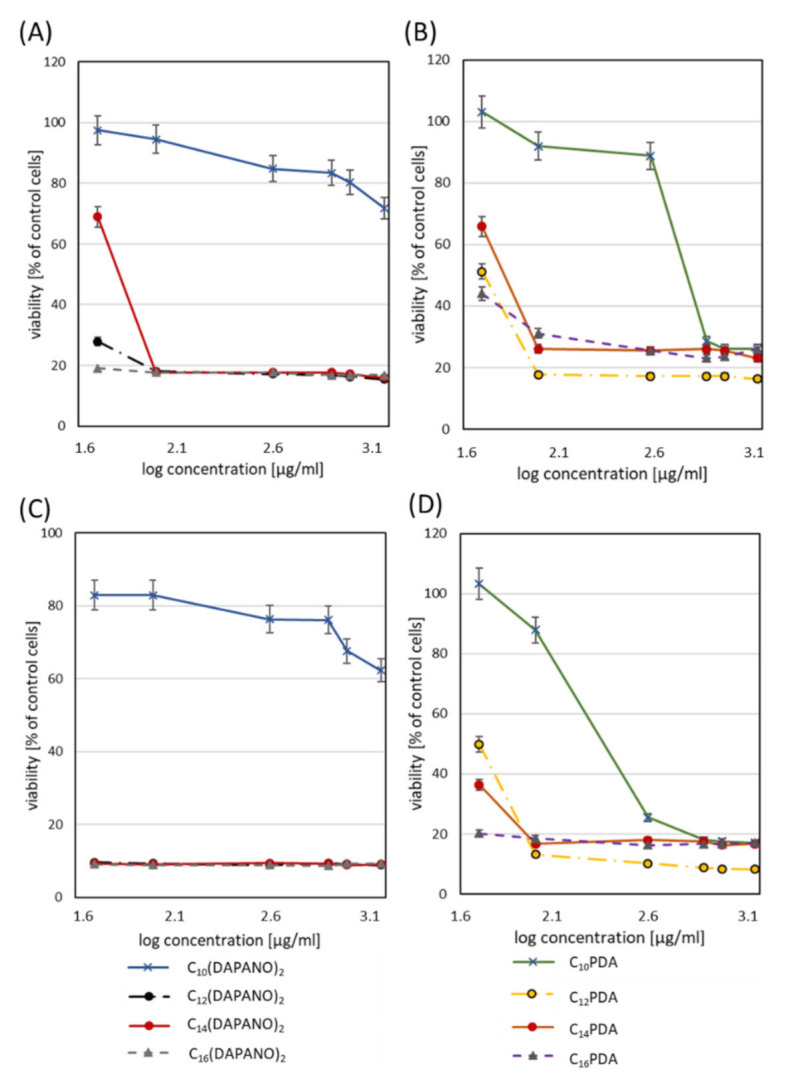
Evaluation of primary fibroblasts (HGFs) viability after 24 (**A**,**B**) and 48 h incubation (**C**,**D**) with C_n_(DAPANO)_2_ and C_n_PDA surfactants in the range 50–2000 µg/mL.

**Figure 9:**
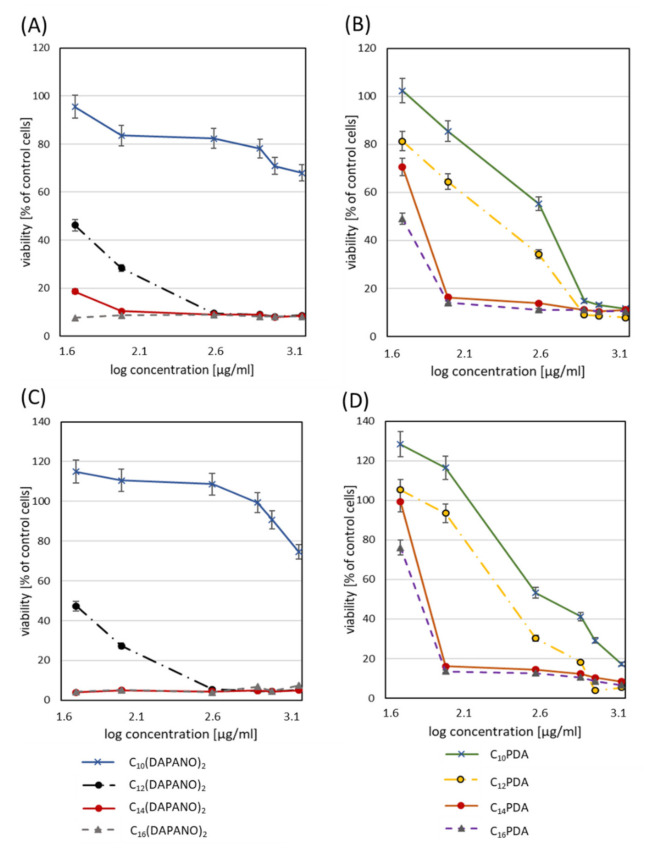
Evaluation of human keratinocytes viability after 24 (**A**,**B**) and 48 h incubation (**C**,**D**) with C_n_(DAPANO)_2_ and C_n_PDA surfactants in the range 50–2000 µg/mL.

In this part of our work, a human normal cell model was used. Keratinocytes and fibroblasts were selected for experiments because they allow for the determination of surfactant interactions with skin and mucosa cells. HaCaT and HGFs cells are thus an interesting model to predict surfactant toxicity after superficial skin or mucosal application. IC_50_ is presented in Table 2, calculated after 24 and 48 h incubation. Cell viability percentage is plotted as a function of surfactant concentration (semi-log plot) (Figure 8 and Figure 9). In all cases, decreased cell proliferation was observed with the longer incubation time with surfactants. Less cytotoxicity occurred in PDA surfactants. In human keratinocytes, IC_50_ for 24 h resulted in concentrations higher than 0.03 g/L, and for 48 h, 50% of the cytotoxic effect was observed for 0.0415 g/L (C_10_-PDA). The proliferation studies proved the decreased toxicity for C_10_(DAPANO)_2_ in the case of normal keratinocytes (IC_50_24 h = 0.098 g/L and IC_50_48 h = 0.097 g/L). In the case of human fibroblasts, a low cytotoxicity level was observed by the selected surfactants: C_14_(DAPANO)_2_ (IC_50_24 h = 0.0685 g/L), C_10_PDA (IC_50_24 h = 0.0779 g/L and IC_50_48 h = 0.0387 g/L), and C_14_PDA (IC_50_24 h = 0.0699 g/L). Table 2 shows the viability of both cell lines evaluated for CMC after 24 and 48 h. We can found that our experiments showed in most cases cytotoxicity near the CMC or below what refers to the good ability of micelle formation. Our study indicates that the C_n_PDA surfactants are safe in CMC concentration in bath-treated cell lines. However, C_n_(DAPANO)_2_ type surfactants induce less cytotoxicity below CMC, which can suggest a better potential for application in emulsion form. Other studies are mainly focused on anticancer activity and cytostatic delivery in the tumor cells; however, some authors have applied cytotoxicity studies. They have indicated that there is no connection between CMC and cytotoxicity in the case of surfactants tested by the authors [48]. It seems that a low CMC with a high IC_50_ value will be optimal for micelle solubilization and surfactant safety in biological systems. Kyadarkunte et al. evaluated the effects of the fatty acid chain length of acylglutamate (amino acid-based surfactant) on HaCaT cells. The authors observed low cytotoxicity and that the shorter the fatty acid chain length of the acylglutamate surfactants, the less the cytotoxic effect was induced on the HaCaT cell line [49]. In our study, we can indicate the dependence on the carbon numbers. Our intention was the overall objective of developing a biologically safe system mainly for normal but also pathological cells. The dicephalic surfactants presented here are intended to also be safe for skin and mucosa cells and natural microbial flora. Most of the available data are mainly involved in drug delivery in tumor cells with nanocarrier-based surfactants [50].

**Figure 10:**
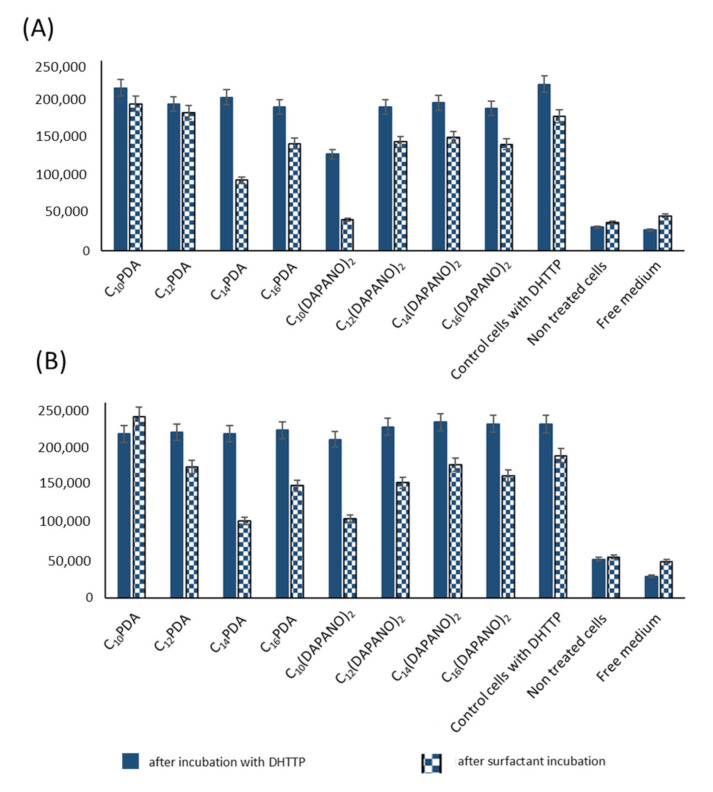
The evaluation of the antioxidant potential of surfactants in (**A**) primary fibroblasts and (**B**) human keratinocytes after ROS induction by DHTTP. Surfactants’ concentration was applied in the CMC range.

The ROS evaluation results are presented in Figure 10. As we can observe, the DHTTP solution provoked a high and significant increase of radicals in normal keratinocytes and fibroblasts. After surfactant addition in CMC concentration, we could observe a decrease in the radical level. However, the effect is synergistically caused by natural blanking fluorescence signals and by the antioxidative properties of applied surfactants. The most significant decrease of fluorescence level was induced by C_14_PDA (linear analog of C_14_(DAPANO)_2_); then, a similar decrease was observed in the case of C_16_PDA and C_10_(DAPANO)_2_ in both treated cell lines. Surfactants C_14_(DAPANO)_2_ and C_16_(DAPANO)_2_ also induced a not significant decrease in radical level. Some authors indicate a strong peroxidative effect of surfactants [51], which can be excluded for applications in carriers for drug delivery. Some reports have demonstrated that low concentrations of cationic surfactants can induce apoptosis in different mammalian cell types [52,53]. Antiradical properties can be the most significant feature in surfactant selection in future biological applications.

## 3. Conclusions

In this investigation, the aim was to assess the antiradical activity of monocephalic 3-alkanoylamino)propyldimethylamine-N-oxides (C_n_PDA) and dicephalic N,N-bis[3,3′-(dimethylamino)propyl]alkylamide di-N-oxides [C_n_(DAPANO)_2_]. This was tested against two stable radicals, namely DPPH• and GO•, using electron paramagnetic resonance (EPR) and UV-vis spectroscopy. We showed that both C_n_PDA and C_n_(DAPANO)_2_ surfactants scavenge radicals and thus can protect biological systems as well as cosmetic and pharmaceutical formulations. The determination of the second-order rate constants k_2_ revealed that the two head-single tail C_n_(DAPANO)_2_ surfactants neutralize radicals faster than their linear counterparts. We also found that the ability of the N-oxide surfactants to scavenge radicals increases with the length of the aliphatic chain. The computational findings reported here shed light on the mechanism of antiradical action. By the inspection of the predicted bond dissociation enthalpies, ionization potentials, and Gibbs free energies, we identified hydrogen atom transfer (HAT) as the most probable mechanism. In in vitro studies, longer incubation time with surfactants leads to decreased cell proliferation. C_n_PDA surfactants occurred as less cytotoxic against tested cell lines. In most cases, cytotoxicity was near the CMC or below. In ROS evaluation, after adding the surfactants in CMC concentration, the level of radicals decreased. The best results were obtained after using C_14_PDA and similar in the case of C_16_PDA and C_10_(DAPANO)_2_ in both treated cell lines.

All in all, this study provides strong empirical confirmation that the N-oxide surfactants have antiradical activity. In combination with their antimicrobial and immunomodulatory properties and a broad range of profound aggregation behavior, this makes them a multipurpose tool for biomedical uses.

## 4. Materials and Methods

### 4.1. Materials

C_n_(DAPANO)_2_ were synthesized as described in the patent [18]. Elemental analyses (Vario EL III CHNS analyzer) and ^1^H NMR spectrum (CDCl_3_, 500 MHz) were carried out for C_n_(DAPANO)_2_ series and are presented as follows:

**C_10_(DAPANO)_2_** M 373.65: Anal. Calc. (%) for C_20_H_43_N_3_O_3_, C, 64.28; H, 11.60; N 11.25. Found: C, 64.26; H, 11.55; N 11.25; ^1^H NMR (CDCl_3_, 500 MHz): 0.82 (t, 3H, *^3^J_HH_* = 6.6 Hz, ***CH_3_***(CH_2_)_6_CH_2_CH_2_CON-); 1.22 (m, 12H, CH_3_(***CH_2_***)_6_CH_2_CH_2_CON-); 1.57 (k, 2H CH_3_(CH_2_)_6_***CH_2_***CH_2_CON-); 1.60–1.68 (k, 4H-N[(CH_2_***CH_2_***CH_2_N(CH_3_)_2_)_2_]); 2.18–2.20 (s, 12H, -N[(CH_2_CH_2_CH_2_N(***CH_3_***)_2_)_2_]); 2.21 (t, 2H, -CH_3_(CH_2_)_6_CH_2_***CH*_2_**CON-); 2.24–2.28 (t, 4H, -N[(CH_2_CH_2_***CH_2_***N(CH_3_)_2_)_2_]); 3.22–3.30 (t, 4H -N[(***CH_2_***CH_2_CH_2_N(CH_3_)_2_)_2_]);

**C_12_(DAPANO)_2_** M 401.19: Anal. Calc. (%) for C_22_H_47_N_3_O_3_, C, 65.86; H, 11.81; N 10.47. Found: C, 65.78; H, 11.85; N 10.45; ^1^H NMR (CDCl_3_, 500 MHz): 0.86 (t, 3H, *^3^J_HH_* = 6.6 Hz, ***CH_3_***(CH_2_)_8_CH_2_CH_2_CON-); 1.25 (m, 16H, CH_3_(***CH_2_*)**_8_CH_2_CH_2_CON-); 1.62–1.71 (k, 4H, -N[(CH_2_***CH_2_***CH_2_N(CH_3_)_2_)_2_]); 1.64 (k, 2H, CH_3_(CH_2_)_8_***CH_2_***CH_2_CON-); 2.17–2.21 (s, 12H, -N[(CH_2_CH_2_CH_2_N(***CH_3_***)_2_)_2_]); 2.25 (t, 2H, CH_3_(CH_2_)_8_CH_2_***CH_2_***CON-); 2.25–2.30 (t, 4H, -N[(CH_2_CH_2_***CH_2_***N(CH_3_)_2_)_2_]); 3.27–3.33 (t, 4H, -N[(***CH_2_***CH_2_CH_2_N(CH_3_)_2_)_2_]);

**C_14_(DAPANO)_2_** M 429.21: Anal. Calc. (%) for C_24_H_51_N_3_O_3_, C, 67.16; H, 11.98; N 9.79. Found: C, 67.13; H, 11.86; N 9.75; ^1^H NMR (CDCl_3_, 500 MHz): 0.85 (t, 3H, *^3^J_HH_* = 6.6 Hz, ***CH_3_***(CH_2_)_10_CH_2_CH_2_CON-); 1.24 (m, 20H, CH_3_(***CH_2_***)_10_CH_2_CH_2_CON-); 1.62 (k, 2H, CH_3_(CH_2_)_10_***CH_2_***CH_2_CON-); 1.62–1.70 (k, 4H, -N[(CH_2_***CH_2_***CH_2_N(CH_3_)_2_)_2_]); 2.18–2.21 (s, 12H, -N[(CH_2_CH_2_CH_2_N(***CH_3_***)_2_)_2_]); 2.24 (t, 2H, CH_3_(CH_2_)_10_CH_2_***CH_2_***CON-); 2.24–2.30 (t, 4H, -N[(CH_2_CH_2_***CH_2_***N(CH_3_)_2_)_2_]); 3.28–3.33 (t, 4H, -N[(***CH_2_***CH_2_CH_2_N(CH_3_)_2_)_2_]);

**C_16_(DAPANO)_2_** M 457.23: Anal. Calc. (%) for C_26_H_55_N_3_O_3_, C, 68.26; H, 12.12; N 9.19. Found: C, 68.20; H, 12.06; N 9.05; ^1^H NMR (CDCl_3_, 500 MHz): 0.86 (t, 3H, *^3^J_HH_* = 6.6 Hz, ***CH_3_***(CH_2_)_12_***CH_2_***CH_2_CON-); 1.25 (m, 24H, CH_3_(***CH_2_***)_12_CH_2_CH_2_CON-); 1.59 (k, 2H, CH_3_(CH_2_)_12_***CH_2_***CH_2_CON-); 1.64–1.71 (k, 4H -N[(CH_2_***CH_2_***CH_2_N(CH_3_)_2_)_2_]); 2.17–2.20 (s, 12H, -N[(CH_2_CH_2_CH_2_N(***CH_3_***)_2_)_2_]); 2.21–2.30 (t, 4H-N[(CH_2_CH_2_***CH_2_***N(CH_3_)_2_)_2_]); 2.25 (t, 2H, CH_3_(CH_2_)_12_CH_2_***CH_2_***CON-), 3.27–3.32 (t, 4H-N[(***CH_2_***CH_2_CH_2_N(CH_3_)_2_)_2_]);

C_n_PDA were synthesized as described in [19]. Elemental analyses (Vario EL III CHNS analyzer) and ^1^H NMR spectrum (CDCl_3_, 500 MHz) were carried out for C_n_PDA series and are presented as follows:

**C_10_PDA** M 272.15: Anal. Calc. (%) for C_15_H_32_N_2_O_2_, C, 66.13; H, 11.84; N, 10.28; ***CH_3_***(CH_2_)_6_CH_2_CH_2_- 0.84 (t, 3H, *^3^J_HH_* = 6.51 Hz), CH_3_(***CH_2_***)_6_CH_2_CH_2_- 1.22 (m, 12H), CH_3_(CH_2_)_6_***CH_2_***CH_2_- 1.58 (m, 2H),CH_3_(CH_2_)_6_CH_2_***CH_2_***- 2.16 (m, 2H), -CO***NH*** 8.54 (bd, 1H), -CONH***CH_2_***CH_2_ CH_2_N 3.95 (t, 2H), -CONHCH_2_***CH_2_*** CH_2_N 2.27 (t, 2H), -CONHCH_2_ CH_2_***CH_2_*** N 3.39 (m, 2H), -N(***CH_3_***)_2_ 3.54 (s, 6H);

**C_12_PDA** M 300.48: Anal. Calc. (%) for C_17_H_36_N_2_O_2_, C, 67.95; H, 12.08; N, 9.32; **CH_3_**(CH_2_)_8_CH_2_CH_2_- 0.85 (t, 3H, *^3^J_HH_* = 6.51 Hz), CH_3_(***CH_2_***)_8_CH_2_CH_2_- 1.24 (m, 16H), CH_3_(CH_2_)_8_ ***CH_2_***CH_2_- 1.50 (m, 2H), CH_3_(CH_2_)_8_CH_2_***CH_2_***- 2.18 (m, 2H),-CO***NH*** 8.52 (bd, 1H), -CONH***CH_2_***CH_2_ CH_2_N 3.75 (t, 2H), -CONHCH_2_***CH_2_*** CH_2_N 2.27 (t, 2H), -CONHCH_2_ CH_2_**CH_2_** N 3.45 (m, 2H), -N(***CH_3_***)_2_ 3.52 (s, 6H);

**C_14_PDA** M 328.53: Anal. Calc. (%) for C_19_H_40_N_2_O_2_, C, 69.46; H, 12.27; N, 8.53; **CH_3_**(CH_2_)_10_CH_2_CH_2_- 0.84 (t, 3H, *^3^J_HH_* = 6.52 Hz), CH_3_(***CH_2_***)_10_CH_2_CH_2_- 1.34 (m, 20H), CH_3_(CH_2_)_10_ ***CH_2_***CH_2_- 1.57 (m, 2H), CH_3_(CH_2_)_10_CH_2_***CH_2_***- 2.15 (m, 2H), -CO***NH*** 8.43 (bd, 1H), -CONH***CH_2_***CH_2_ CH_2_N 3.74 (t, 2H), -CONHCH_2_***CH_2_*** CH_2_N 2.31 (t, 2H), -CONHCH_2_ CH_2_***CH_2_*** N 3.49 (m, 2H), -N(***CH_3_***)_2_ 3.32 (s, 6H);

**C_16_PDA** M 356.19: Anal. Calc. (%) for C_21_H_44_N_2_O_2_, C, 70.73; H, 12.44; N, 7.86; **CH_3_**(CH_2_)_12_CH_2_CH_2_- 0.86 (t, 3H, *^3^J_HH_* = 6.51 Hz), CH_3_(***CH_2_***)_12_CH_2_CH_2_- 1,31 (m, 24H), CH_3_(CH_2_)_12_ ***CH_2_***CH_2_- 1.58 (m, 2H), CH_3_(CH_2_)_12_CH_2_***CH_2_***- 2.22 (m, 2H),-CO***NH*** 8.50 (bd, 1H), -CONH***CH_2_***CH_2_ CH_2_N 3.92 (t, 2H), -CONHCH_2_***CH_2_*** CH_2_N 2.41 (t, 2H), -CONHCH_2_ CH_2_***CH_2_*** N 3.41 (m, 2H), -N(***CH*_3_**)_2_ 3.54 (s, 6H).

Organic solvents used in the synthesis of surfactants were reagent grade and were used without further purification. Water used in experiments was doubly distilled and purified using a Millipore Milli-Q purification system (Bedford, MA, USA). Ethanol for spectroscopic experiments was purchased from J.T. Baker (99.5%); 2,2-diphenyl-1-picrylhydrazyl (DPPH•) and galvinoxyl (GO•) radicals were purchased from Sigma-Aldrich (St. Louis, MO, USA).

### 4.2. Tension Measurements

Equilibrium surface tension measurements for C_n_PDA were performed using a Krüss K12 microprocessor tensiometer (Krüss, Hamburg, Germany) equipped with a du Nouy Pt−Ir ring (resolution ± 0.01 mN/m). The surface tension was obtained as the arithmetic means of the values received from two independent runs; the data were reproducible within ±0.2 mN m^−1^. Sets of experiments were taken at intervals until no significant change in the surface tension occurred. All of the surface tension measurements were performed at 295 ± 0.1 K. The absence of a minimum in the isotherm curves near CMC was evidence of the purity of the studied surfactants.

### 4.3. Dynamic Light Scattering (DLS)

The hydrodynamic diameter for C_n_PDA was determined by the DLS method. The measurements were performed using a Nano Series Zetasizer from Malvern Instruments (UK) with a detection angle of 173°, equipped with a He−Ne laser (632.8 nm) and an ALV 5000 multibit multitau autocorrelator. Before the measurements, the samples were filtered through a filter (with a pore size of 0.2 μm) directly into the optical cell to remove any impurities. All of the DLS measurements were performed at 298 ± 0.1 K and at a surfactant concentration a hundred times higher than the critical micelle concentration (CMC). Each value was obtained as the average of three runs with at least 10 measurements. The DTS software (Nano) was used to evaluate the data.

### 4.4. Electron Paramagnetic Resonance (EPR) Spectroscopy

The antiradical properties were studied by EPR using a Bruker ELEXYS E500 spectrometer equipped with an NMR gaussmeter and frequency counter. A microwave power of 10 mW, a modulation frequency of 100 kHz, a modulation amplitude of 1 G, a time constant of 82 ms, and a conversion time of 164 ms were adopted. The antiradical properties were evaluated against two model radicals: 2,2-diphenyl-1-picrylhydrazyl (DPPH•) and galvinoxyl (GO•). The measurements were done at 298 K in ethanol solutions containing DPPH^•^ (C = 2.0 mM) or GO^•^ (2.0 mM) and C_n_PDA (C = 100 CMC) or C_n_(DAPANO)_2_ (C = 100 CMC). The reference samples contained only the model radicals. The samples were sealed in capillary tubes and placed inside a standard EPR quartz tube for measurements.

### 4.5. UV−Vis Measurements

The measurements were done under deaerated conditions at 298 K in ethanol solutions (2 mL in a 10 mm quartz cuvette) containing DPPH^•^ (C = 2.2 × 10^−4^ M) and a surfactant at each of five different concentrations, namely C_1_ = 0.12 M, C_2_ = 0.09 M, C_3_ = 0.06 M, C_4_ = 0.03 M, and C_5_ = 0.012 M. UV−vis spectral changes associated with the undergoing reaction were monitored at 516 nm, using a Varian Cary 50Conc UV−visible Spectrophotometer. The following equations of the calibration curve were used to calculate DPPH^•^ concentrations in the reaction system: Abs = 4.0189 × C[mM] + 0.00813 (R^2^ = 0.9998). The rate of DPPH^•^ scavenging reaction was determined using pseudo-first-order kinetics [54,55] (the concentration of C_n_PDA or C_n_(DAPANO)_2_ was maintained at more than 54-fold excess of the radical concentration); further details are given as Supporting Information.

### 4.6. Theoretical Calculations

Unless otherwise noted, the theoretical calculations were conducted with the ORCA 4.1/4.2 suite of programs [56,57] and are summarized in Table S4. The geometry optimizations were carried out at the DFT level using the gradient-corrected BP86 functional [58,59], which provides accurate molecular structures [60,61]. Each of the stationary points was fully characterized as a true minimum through a vibrational analysis. On these molecular structures, single-point calculations were carried out at the DFT level with the hybrid B3LYP [62,63,64] and M06-2X [65] and double hybrid B2PLYP [66] approximations as well as at the ab initio level with the DLPNO-CCSD(T) method, which was designed to make the coupled-cluster (CC) theory broadly applicable [67,68,69]. The open-shell DLPNO-CCSD calculations were carried out using the reference determinants build from quasi-restricted orbitals (QROs) from the unrestricted Hartree–Fock (UHF) calculations [70]. QROs should closely resemble the restricted open-shell Hartree–Fock (ROHF) orbitals [71]. The perturbative triples correction was computed using the iterative approach to the triples amplitudes [72]. The validity of the DLPNO-CCSD(T) results was assessed using the T1 diagnostic parameter. The observed values of T1 were always noticeably lower than 0.02. The bond dissociation enthalpies (BDE), ionization potentials (IP), and Gibbs free energies (ΔG) were calculated at 298 K with all these theoretical methods but using the zero point, thermal (vibrational, rotational, and translational) and entropy corrections computed at the BP86 level. In all these calculations the conductor-like polarizable continuum model (CPCM) [73] was used to cover the solvent effects (for DLPNO-CCSD(T) only in the reference determinants). In the DFT calculations, the resolution of the identity approximation [74,75,76] was used, and the dispersion correction was added; double-hybrid functionals already account for parts of the dispersion interaction; hence, for B2PLYP, the parameter S_6_ = 0.64 was used [77,78]. In all the DFT calculations, the def2-TZVP basis set was employed with an appropriate auxiliary basis set (def2/J) [79,80,81]. The DLPNO-CCSD(T) calculations were conducted in concert with the cc-pVTZ basis set combined with an appropriate auxiliary basis set for correlation calculations (cc-pVTZ/C) [82,83].

The time-dependent DFT (TD-DFT) computations were carried out with the functional B3LYP and B2PLYP along with the def2-TZVP basis set. To analyze these results, the natural transition orbitals (NTOs) were used [29].

The values of LogP were predicted at the DFT level using the Amsterdam Density Functional (ADF) program [84] using the COSMO-RS module [85]. These calculations were carried out at the BP86/TZP theory level.

### 4.7. Antioxidative Properties—ROS Evaluation

Antioxidative properties of the tested surfactant were evaluated. To induce ROS production, a DPPH solution was prepared (C = 2.55 × 10^−3^ mol/dm3). Normal fibroblasts and keratinocytes were seeded on flat bottom 96-well tissue culture plates at a density of 10^4^ cells/well and allowed to grow in a CO_2_ incubator at 37°C overnight. Then, culture medium was removed and 200 µL of DPPH solution in DMEM was added to the plate and left for 1 h in a CO_2_ incubator at 37 °C. After incubation, DPPH solution was removed and ROS production was determined using DCF assay (Life Technologies, Warsaw, Poland). The DCF assay was conducted with 6-carboxy-2′,7′-dichlorodihydrofluorescein diacetate 2′,7′-dichlorofluorescein (carboxy-H_2_DCFDA). After deacetylation of H_2_DCFDA to H_2_DCF or carboxy-H_2_DCFDA to carboxy-H_2_DCF, cellular ROS oxidize H_2_DCF and carboxy-H_2_DCF to 2′,7′-dichlorofluorescein (DCF) and 2′,7′-carboxydichlorofluorescein (carboxy-DCF), respectively. The carboxy-H_2_DCFDA was dissolved in sterile DMSO to generate a stock solution of 50 µg/mL. For experiments, the stock solution of carboxy-H_2_DCFDA was brought to room temperature in the dark and then diluted in a cell culture medium without FBS. After washout of the incubation medium from cells with PBS, the reagent was added to cell culture to a final concentration of 10 µM and for 30 min was incubated at 37 °C in darkness. After incubation, excitation wavelength of 495 nm and emission wavelength of 530 nm were used by a multiwell scanning spectrophotometer (EnSpire Perkin Elmer, Krakow, Poland). In the next stage, surfactant solutions in CMC concentrations were added into cells after 1 h incubation with DHTTP solution. Cells were incubated, and ROS were detected 30 min by a multiwell scanning spectrophotometer (EnSpire Perkin Elmer, Poland).

### 4.8. Cell Culture

HaCaT (immortalized human keratinocytes from histologically normal skin) cell line was obtained from the CLS Cell Lines Service GmbH (Eppelheim, Germany). HGFs (human gingival fibroblasts) derive from primary cell culture. The cells were isolated according to the procedure described by Saczko et al. [86] and patented by Saczko et al. [87]. Cells were grown in plastic cell culture flasks in Dulbecco’s Modified Eagle’s Medium, supplemented with fetal bovine serum (FBS, Lonza, Morrisville, NC, USA), with 100 IU/mL penicillin, 50 mg streptomycin, and 125 μg amphotericin B (Sigma). The cells were maintained in a humidified atmosphere at 37 °C and 5% CO_2_. The cells were regularly maintained by systematic passaging. For experimental purposes, the cells were removed by trypsinization (0.25% Trypsin-EDTA, Sigma).

### 4.9. MTT Cell Viability Assay

MTT (Sigma) test was used to determine the viability of HaCaT and HGFs cells. The MTT assay was used to test mitochondrial metabolic functioning. Cells were seeded on flat bottom 96-well tissue culture plates at a density of 10^4^ cells/well and allowed to grow in a CO_2_ incubator at 37 °C overnight. In the case of these studies, the culture medium was removed, surfactant solutions were added, and the cells were exposed for 24 and 48 h to compounds at doses ranging from 50 to 2000 μg/mL. After incubation, MTT assay was applied according to the manufacturer protocol. The absorbance was measured at 570 nm using a multiwell plate reader (EnSpire Multimode Reader, Perkin Elmer). Each experiment was performed in a few independent repetitions. Mean values and standard deviations of all results were calculated. The final results were expressed as the percentage of mitochondrial function relative to untreated control cells.

### 4.10. Statistical Analysis

Unless otherwise indicated, all the data are mean values ± SD calculated from at least three independent experiments. The Student’s *t* test was used to check the significant level between independent variables. The level of significance was set to *p* < 0.05.

## Figures and Tables

**Table 1:** Selected properties of the examined surfactants: critical micelle concentrations (CMC), hydrodynamic radius (R_H_), the degree of hydrophobicity from the COSMO-RS calculations [defined as the logarithm of the octanol-water partition coefficient (logP)].

	Abbreviations	R	CMC [M]	R_H_ [nm]		logP	Lipinski Rule
				DLS	DFT ^b^		
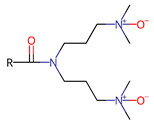	C_10_(DAPANO)_2_	C_9_H_19_	3.0·10^−2 a^	1.75 ^a^	1.79	0.44	Yes
	C_12_(DAPANO)_2_	C_11_H_23_	5.5·10^−3 a^	2.04 ^a^	2.02	1.27	Yes
	C_14_(DAPANO)_2_	C_13_H_27_	8.0·10^−4 a^	2.28 ^a^	2.32	1.97	Yes
	C_16_(DAPANO)_2_	C_15_H_31_	1.5·10^−4 a^	2.38 ^a^	2.46	2.76	Yes
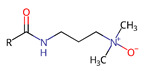	C_10_PDA	C_9_H_19_	7.5·10^−^^3^	1.67	1.75	3.45	Yes
	C_12_PDA	C_11_H_23_	7.0·10^−^^4^	1.96	2.00	4.74	Yes
	C_14_PDA	C_13_H_27_	8.0·10^−^^5^	2.19	2.11	6.05	Yes
	C_16_PDA	C_15_H_31_	8.5·10^−^^6^	2.27	2.51	7.32	Yes

^a^ Data from ref. [17]. ^b^ Assumed as the distance between the carbon atom of the terminal methyl group and the farthest nitrogen atom of the N-oxide group.

**Table 2:** Evaluation IC_50_ for normal human keratinocytes (HaCaT) and primary fibroblasts (HGFs) and the percentage of viability concentration after 24 and 48 h incubation with surfactants.

Surfactant	IC_50_ [g/L]				Viability [%]			
	HaCaT		HGFs		24 h		48 h	
	24 h	48 h	24 h	48 h	HGF’s	HaCaT	HGF’s	HaCaT
C_10_(DAPANO)_2_	0.098	0.097	0.0345	0.0019	16.2	8.1	9.7	18.2
C_12_(DAPANO)_2_	0.095	0.094	0.0279	0.0012	17.1	9.2	10.8	16.4
C_14_(DAPANO)_2_	0.032	0.017	0.0685	0.00089	17.7	11.3	11.5	4.5
C_16_(DAPANO)_2_	0.029	0.0182	0.0143	0.00076	27.8	14.7	12.3	3.9
C_10_PDA	0.0438	0.0415	0.0779	0.0387	98.6	34.7	20.2	84.8
C_12_PDA	0.0310	0.0188	0.0519	0.0498	55.5	89.4	44.8	115.8
C_14_PDA	0.0481	0.0099	0.0699	0.0016	103.7	81.1	78.2	119.4
C_16_PDA	0.0481	0.0098	0.0441	0.0019	96.9	139.5	81.7	123.1

## Data Availability

Not applicable.

## Supplementary Materials

The following are available online at https://www.mdpi.com/article/10.3390/ijms22158040/s1.
