# Supplementary material for: Antiradical Properties of N-Oxide Surfactants—Two in One
Source: Int J Mol Sci. 2021 Jul 27;22(15):8040. doi: 10.3390/ijms22158040 (PMC8346996; doi:10.3390/ijms22158040)
Supplement: Supplementary file 1 [file ijms-22-08040-s001.zip › ijms-1300878-supplementary.pdf]

Antiradical Properties of N-Oxide Surfactants – Two in One

Agnieszka Lewińska[1*], Julita Kulbacka[2], Marta Domżał[3], Maciej Witwicki[1*]

[1] Faculty of Chemistry, University of Wrocław, Joliot-Curie 14, 50-383 Wrocław, Poland
[2]Department of Molecular and Cellular Biology, Faculty of Pharmacy, Wrocław Medical University, Borowska 211A, 50-367 Wrocław, Poland julita.kulbacka@umed.wroc.pl
[3] Faculty of Biotechnology, University of Wrocław, Joliot-Curie 14a, 50-383 Wrocław, Poland
** Correspondence: agnieszka.lewinska@chem.uni.wroc.pl, maciej.witwicki@chem.uni.wroc.pl*

Supplementary Materials

1 Pseudo-first-order kinetics

Scavenging reaction between *DPPH•* and a surfactant *surf-H*:

$$DPPH\bullet + surf\text{-}H \rightarrow R\text{-}H + surf\bullet \qquad (1)$$

can be described by the following kinetic equation:

$$\frac{d[DPPH\bullet]}{dt} = -k_2[surf\text{-}H][DPPH\bullet] \qquad (2)$$

In the experiments the initial concentration of *DPPH•* in the reaction mixtures was fixed at 0.22 mM for *DPPH•* and *surf-H* was always present in at least 54-fold excess. Hence, [*surf-H*] was approximately constant throughout the entire reaction, so (2) becomes:

$$\frac{d[DPPH\bullet]}{dt} = -k_2[surf\text{-}H]_0[DPPH\bullet] = -k_{obs}[DPPH\bullet] \qquad (3)$$

where $k_{obs} = k_2[surf\text{-}H]_0$. This rate equation (3) has the solution:

$$[DPPH\bullet] = [DPPH\bullet]_0 e^{-k_{obs}t} \qquad (4)$$

$$\ln[DPPH\bullet] = \ln[DPPH\bullet]_0 - k_{obs}t \qquad (5)$$

From (5) the pseudo-first-order constant k_{obs} can be calculated and from the slope of the linear plots of k_{obs} vs. [*surf-H*] the second-order rate constants k_2 can be determined. These plots are shown below.

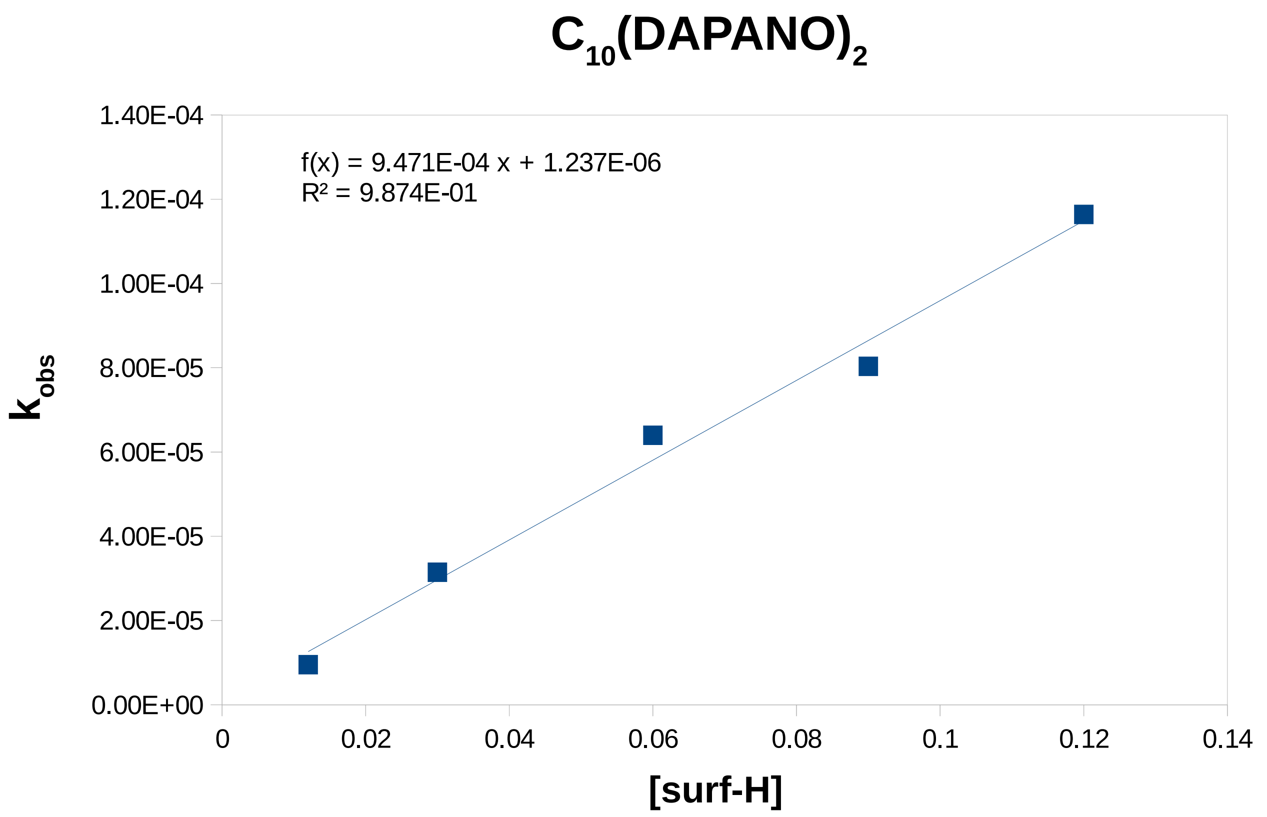

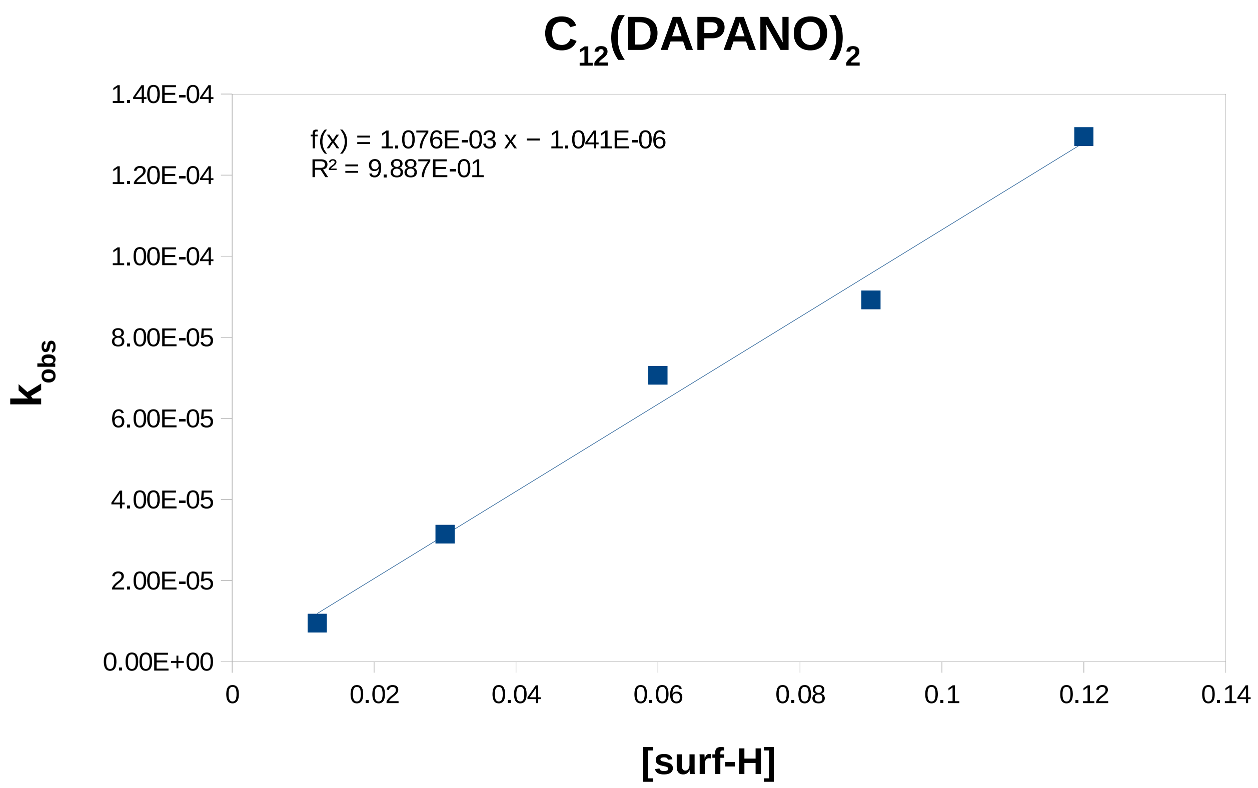

C12(DAPANO)2
f(x) = 1.076E-03 x − 1.041E-06
R² = 9.887E-01
1.40E-04
1.20E-04
1.00E-04
8.00E-05
6.00E-05
4.00E-05
2.00E-05
0.00E+00
0
0.02
0.04
0.06
0.08
0.1
0.12
0.14
kobs
[surf-H]

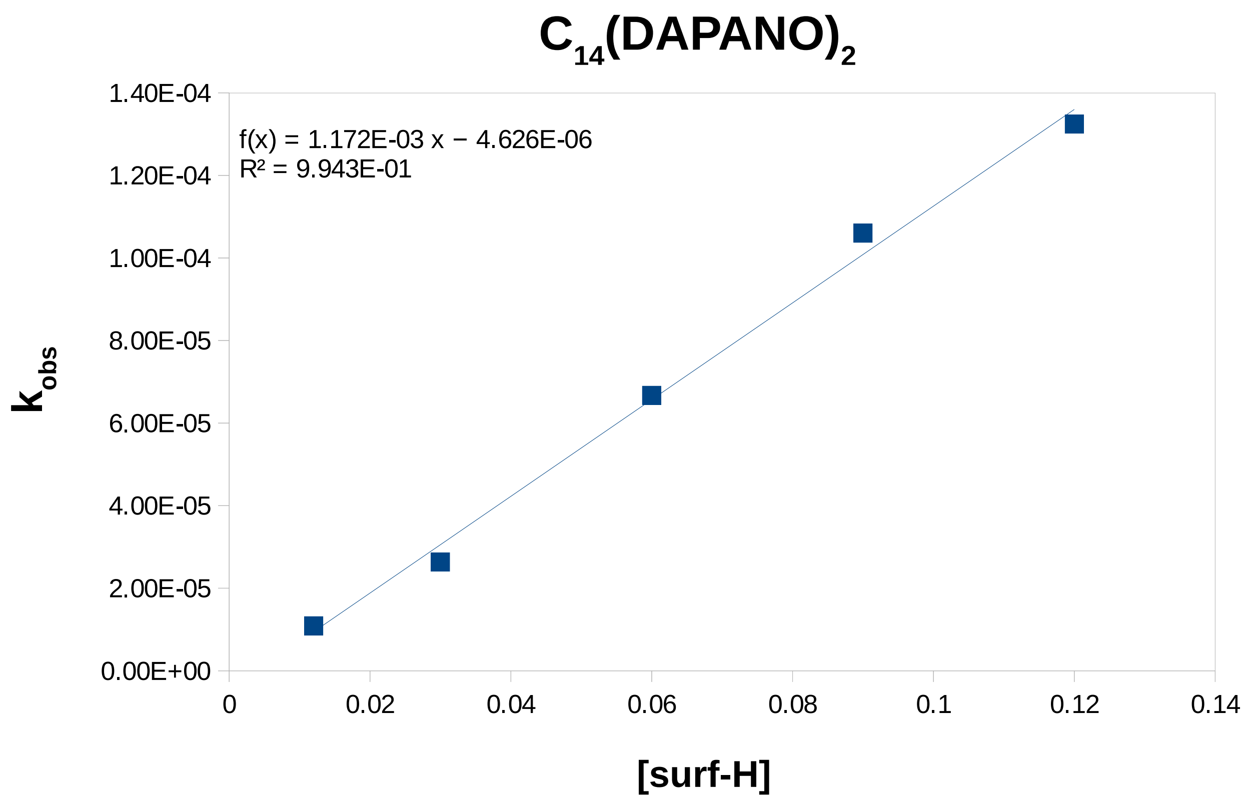

C14(DAPANO)2
f(x) = 1.172E-03 x − 4.626E-06
R² = 9.943E-01
1.40E-04
1.20E-04
1.00E-04
8.00E-05
6.00E-05
4.00E-05
2.00E-05
0.00E+00
0
0.02
0.04
0.06
0.08
0.1
0.12
0.14
kobs
[surf-H]

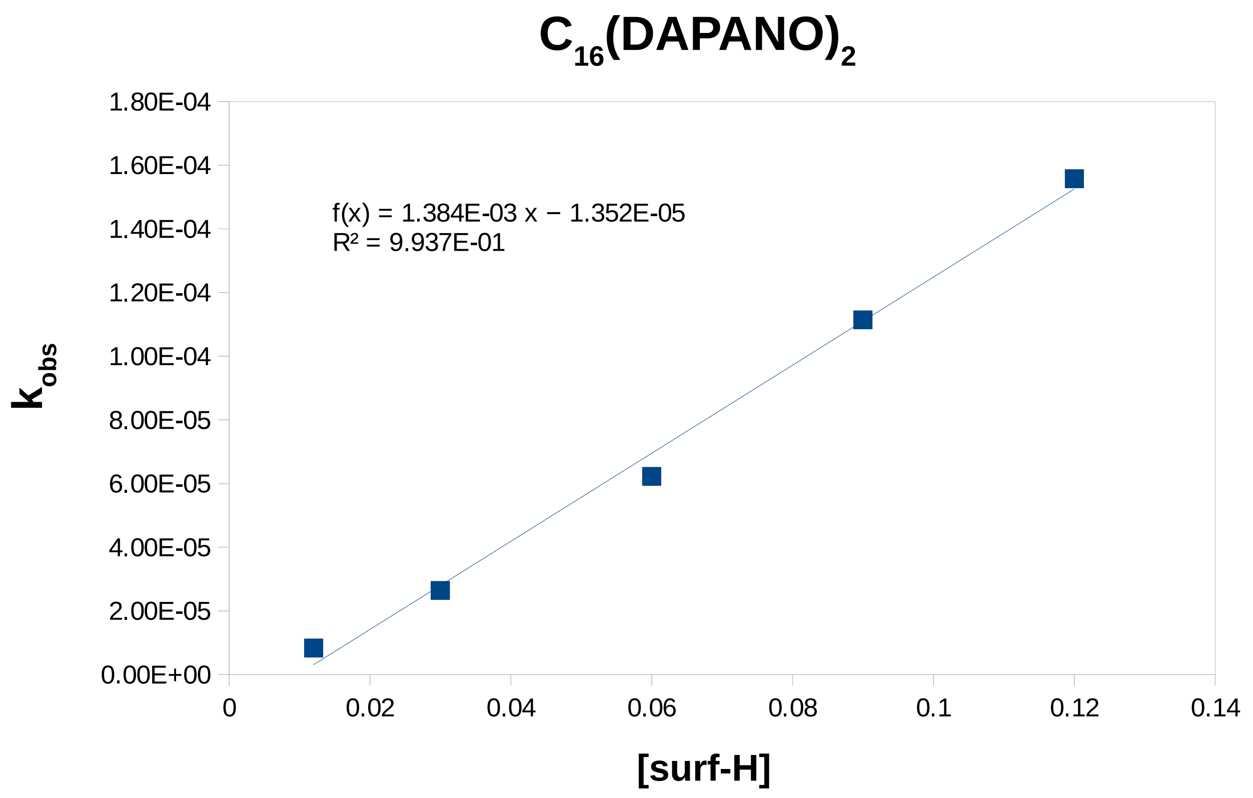
C16(DAPANO)2
f(x) = 1.384E-03 x − 1.352E-05
R² = 9.937E-01
kobs
[surf-H]
1.80E-04
1.60E-04
1.40E-04
1.20E-04
1.00E-04
8.00E-05
6.00E-05
4.00E-05
2.00E-05
0.00E+00
0
0.02
0.04
0.06
0.08
0.1
0.12
0.14

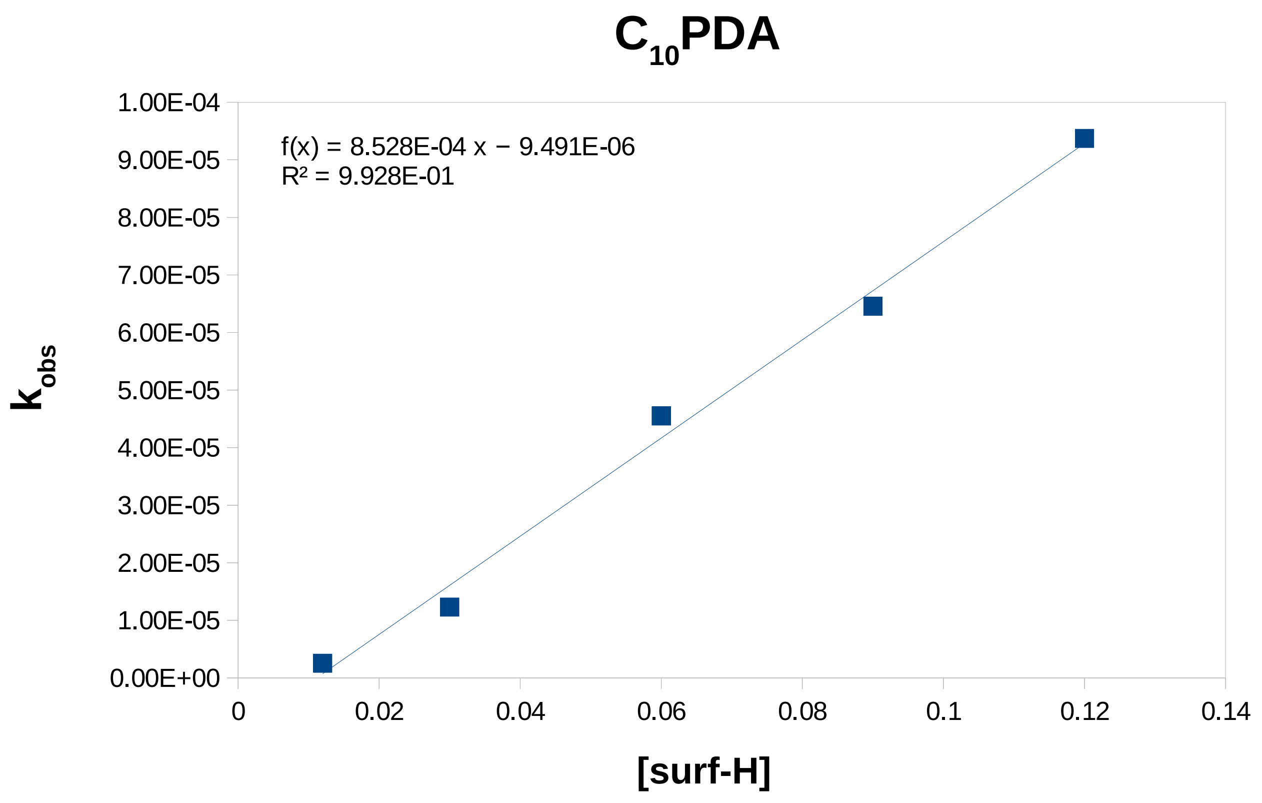
C10PDA
f(x) = 8.528E-04 x − 9.491E-06
R² = 9.928E-01
kobs
[surf-H]
1.00E-04
9.00E-05
8.00E-05
7.00E-05
6.00E-05
5.00E-05
4.00E-05
3.00E-05
2.00E-05
1.00E-05
0.00E+00
0
0.02
0.04
0.06
0.08
0.1
0.12
0.14

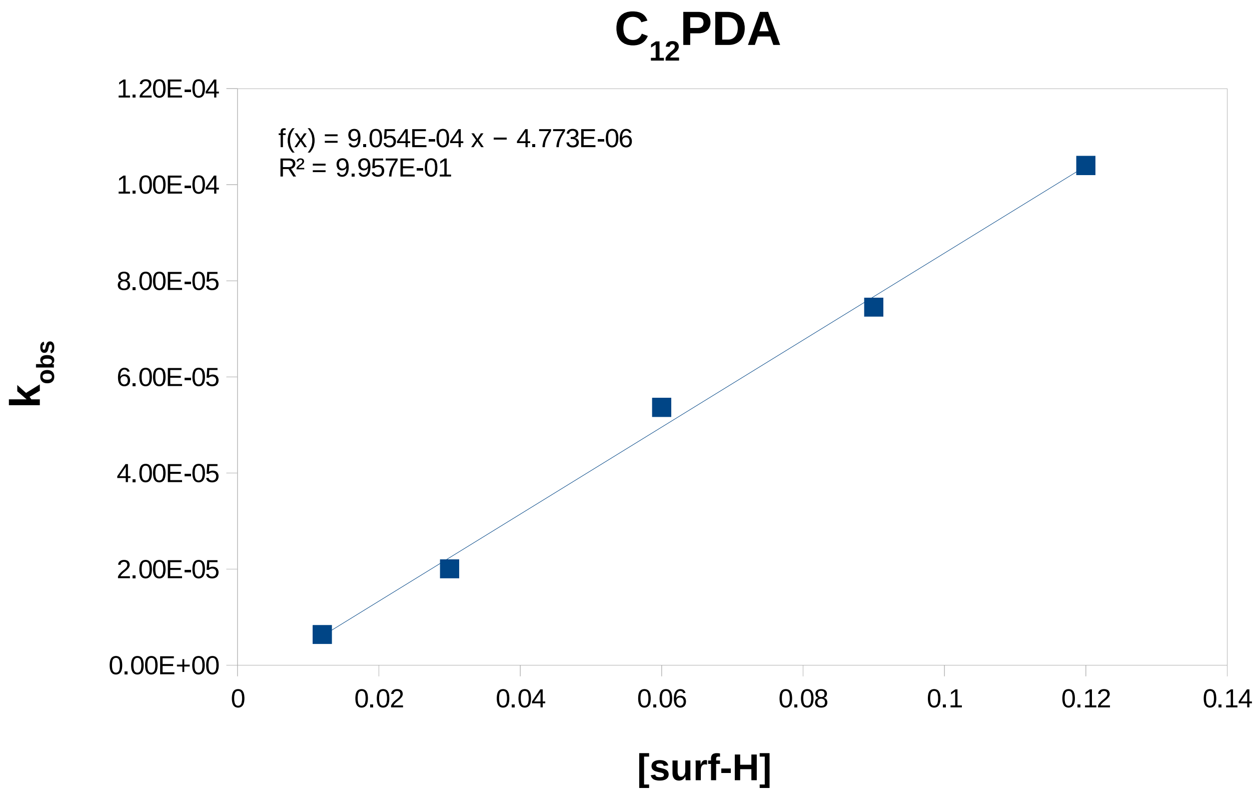

C12PDA
f(x) = 9.054E-04 x − 4.773E-06
R² = 9.957E-01
kobs
[surf-H]
1.20E-04
1.00E-04
8.00E-05
6.00E-05
4.00E-05
2.00E-05
0.00E+00
0
0.02
0.04
0.06
0.08
0.1
0.12
0.14

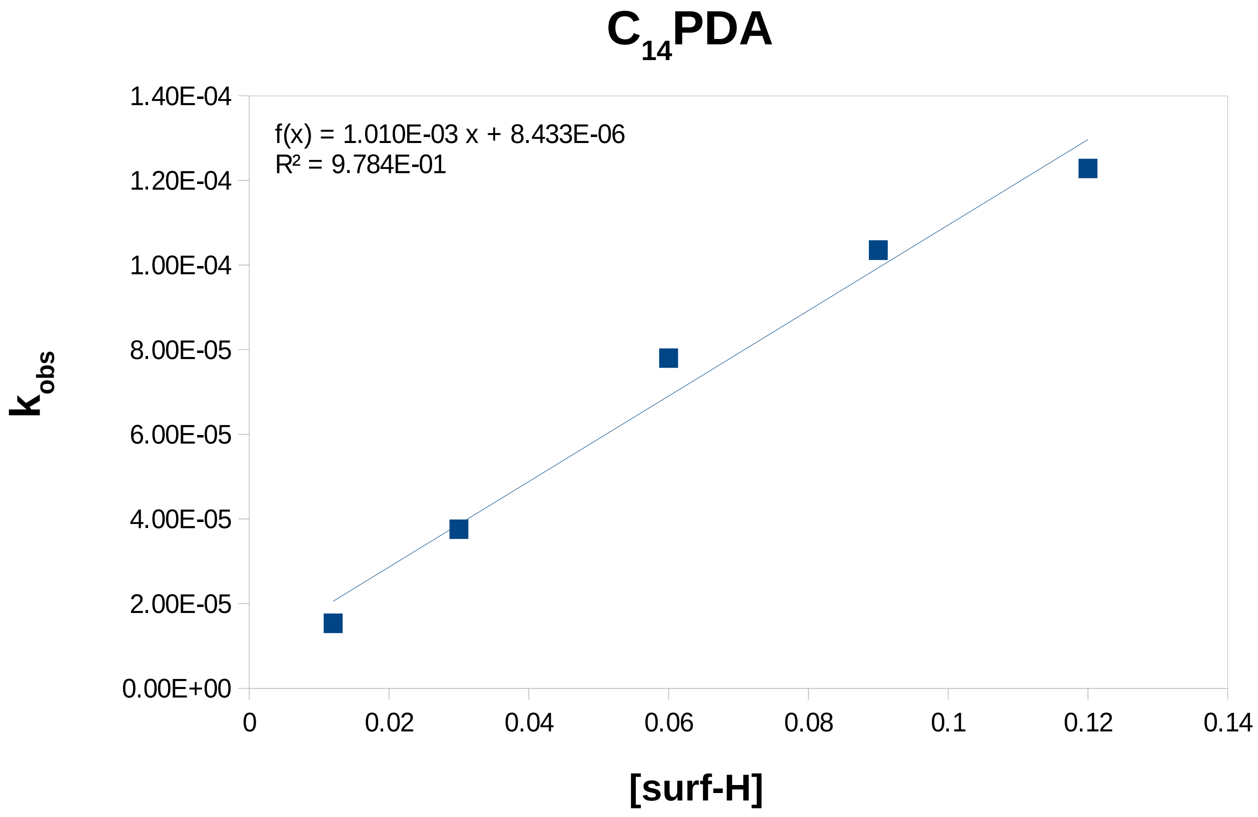

C14PDA
f(x) = 1.010E-03 x + 8.433E-06
R² = 9.784E-01
kobs
[surf-H]
1.40E-04
1.20E-04
1.00E-04
8.00E-05
6.00E-05
4.00E-05
2.00E-05
0.00E+00
0
0.02
0.04
0.06
0.08
0.1
0.12
0.14

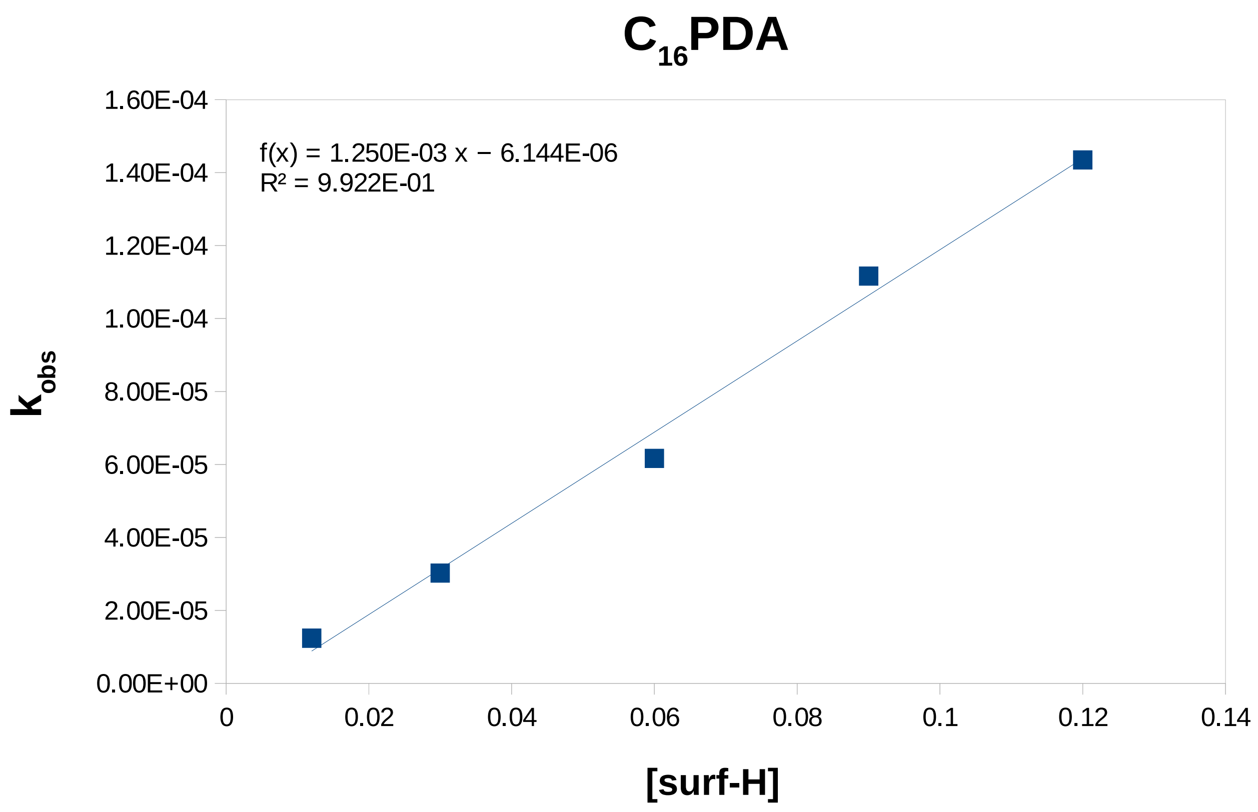

C16PDA
f(x) = 1.250E-03 x − 6.144E-06
R² = 9.922E-01
kobs
1.60E-04
1.40E-04
1.20E-04
1.00E-04
8.00E-05
6.00E-05
4.00E-05
2.00E-05
0.00E+00
0
0.02
0.04
0.06
0.08
0.1
0.12
0.14
[surf-H]

Table S1. Bond dissociation enthalpies (BDE) calculated at 298 K.

		BDE [kcal/mol]			
		**B3LYP**	**M06-2X**	**B2PLYP**	**DLPNO-CCSD(T)**
**C_{10}(DAPANO)$_2$**	**1a**	98.41	102.19	99.41	101.26
	**1a'**	98.48	102.13	99.50	102.15
	**1b**	97.40	101.24	98.43	100.92
	**1b'**	97.69	101.59	98.78	101.33
	**2a**	93.48	97.03	94.68	97.50
	**2b**	94.34	98.07	95.50	98.80
	**3a**	91.51	93.38	92.43	94.39
	**3b**	89.88	93.45	91.50	95.50
	**4a**	91.90	93.12	92.28	93.91
	**4b**	86.81	90.78	88.45	92.47
	**5**	87.39	88.91	87.00	89.26
	**6**	92.77	94.98	93.50	95.85
	**7**	92.42	94.46	93.07	95.60
	**8**	92.55	94.70	93.29	95.05
	**9**	91.85	93.98	92.53	95.13
	**10**	91.91	94.11	92.65	95.22
	**11**	92.45	94.62	93.14	95.00
	**12**	92.25	94.36	92.89	95.51
	**13**	95.48	96.91	95.56	97.54
**C_{10}PDA**	**1**	99.06	102.52	99.93	102.30
	**1'**	99.27	102.73	100.18	102.05
	**2**	97.19	100.11	98.12	100.14
	**3**	94.61	97.36	95.70	98.01
	**4**	87.67	91.24	89.18	91.55
	**0**	103.54	104.87	108.22	105.62
	**5**	95.70	93.45	92.12	94.02
	**6**	94.06	96.68	95.13	96.83
	**7**	94.18	96.36	94.85	97.41
	**8**	94.03	96.47	94.97	97.32
	**9**	106.43	96.84	95.37	109.66
	**10**	94.00	96.20	94.73	97.32
	**11**	94.03	96.25	94.75	97.31
	**12**	93.84	96.01	94.51	96.60
	**13**	97.92	99.34	97.98	99.41
**phenol**		81.85	87.91	85.05	87.47

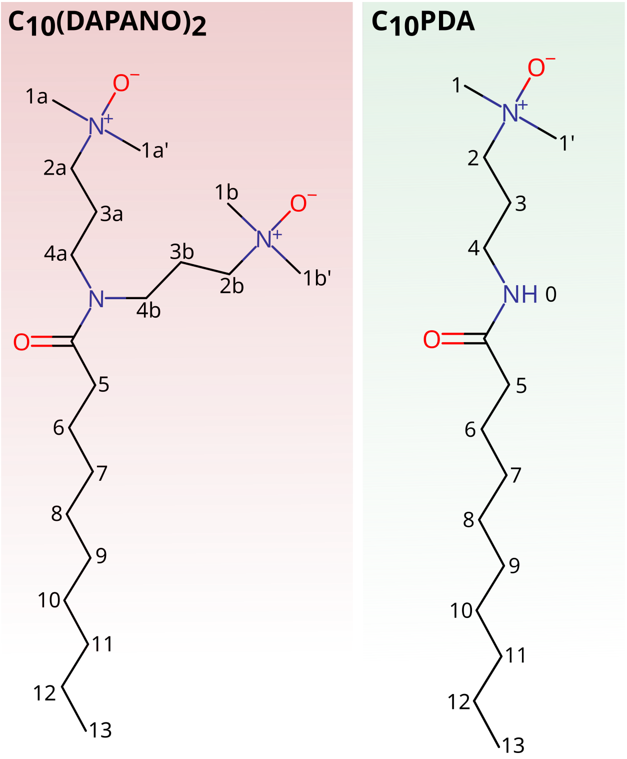

Table S2. Ionisation potentials (IP) calculated at 298 K.

	IP [kcal/mol]			
	**B3LYP**	**M06-2X**	**B2PLYP**	**DLPNO-CCSD(T)**
**$C_{10}(DAPANO)_2$**	143.93	145.87	142.90	147.77
**$C_{10}PDA$**	143.29	145.48	143.15	148.93

Table S3. The Gibbs free energies (ΔG) calculated at 298 K for the hydrogen atom transfer and one-electron transfer process. Fot the hydrogen atom transfer the most stable radical forms of the surfactants were used.

		ΔG [kcal/mol]			
		**B3LYP**	**M06-2X**	**B2PLYP**	**DLPNO-CCSD(T)**
**Hydrogen atom transfer**	***DPPH• + $C_{10}(DAPANO)_2$ → DPPH-H + •$C_{10}(DAPANO)_2$***	10.01	9.26	9.57	11.82
	•OH + $C_{10}(DAPANO)_2$ → H_2O + •$C_{10}(DAPANO)_2$	-29.98	-30.43	-30.32	-29.19
	DPPH• + $C_{10}PDA$ → DPPH-H + •$C_{10}PDA$	22.85	18.32	19.22	21.11
	•OH + $C_{10}PDA$ → H_2O + •$C_{10}PDA$	-17.14	-21.37	-20.67	-19.90
**One-electron transfer**	***DPPH• + $C_{10}(DAPANO)_2$ → $DPPH^-$ + •$C_{10}(DAPANO)_2^+$***	36.71	36.14	36.29	45.56
	•OH + $C_{10}(DAPANO)_2$ → OH^- + •$C_{10}(DAPANO)_2^+$	30.02	31.27	31.11	46.63
	DPPH• + $C_{10}PDA$ → $DPPH^-$ + •$C_{10}PDA^+$	37.01	36.69	37.48	47.67
	•OH + $C_{10}PDA$ → OH^- + •$C_{10}PDA^+$	30.32	31.81	32.30	48.73

Table S4. Summary of the theoretical calculations.

		E [a.u.]					ZPE [au]	TTC [a.u.]	T × S [a.u.]
		**BP86**	**B3LYP**	**M06-2X**	**B2PLYP**	**DLPNO-CCSD(T)**	**BP86**	**BP86**	**BP86**
**C_{10}(DAPANO)$_2$**		-1178.3967962	-1178.3105389	-1177.7566411	-1177.2230630	-1175.8312073	0.6085222	0.0305350	0.0886904
**•C_{10}(DAPANO)$_2$**	**1a**	-1177.7274135	-1177.6386990	-1177.0827860	-1176.5531924	-1175.1572002	0.5947238	0.0306920	0.0898144
	**1a'**	-1177.7271196	-1177.6385926	-1177.0828826	-1176.5530478	-1175.1557787	0.5947260	0.0306854	0.0896094
	**1b**	-1177.7287181	-1177.6403231	-1177.0843096	-1176.5547645	-1175.1577674	0.5947402	0.0306883	0.0895154
	**1b'**	-1177.7281541	-1177.6399260	-1177.0838013	-1176.5542646	-1175.1571582	0.5948988	0.0305823	0.0891883
	**2a**	-1177.7294755	-1177.6442811	-1177.0887398	-1176.5584574	-1175.1609288	0.5953186	0.0278213	0.0849904
	**2b**	-1177.7347870	-1177.6463917	-1177.0905511	-1176.5606204	-1175.1623233	0.5950073	0.0316118	0.0919377
	**3a**	-1177.7328810	-1177.6474058	-1177.0945304	-1176.5620166	-1175.1658641	0.5942699	0.0288521	0.0876458
	**3b**	-1177.7371047	-1177.6503008	-1177.0947206	-1176.5638090	-1175.1643900	0.5932698	0.0301524	0.0885345
	**4a**	-1177.7449691	-1177.6506305	-1177.0987966	-1176.5661011	-1175.1704662	0.5956243	0.0313408	0.0910463
	**4b**	-1177.7447685	-1177.6572696	-1177.1010427	-1176.5707316	-1175.1712977	0.5948959	0.0305951	0.0892603
	**5**	-1177.7405428	-1177.6544013	-1177.1020774	-1176.5710999	-1175.1744583	0.5959751	0.0275693	0.0844222
	**6**	-1177.7290920	-1177.6445061	-1177.0911015	-1176.5594279	-1175.1626421	0.5943021	0.0279312	0.0850409
	**7**	-1177.7289043	-1177.6452591	-1177.0921016	-1176.5603015	-1175.1632357	0.5944594	0.0279599	0.0853008
	**8**	-1177.7287916	-1177.6450101	-1177.0916910	-1176.5599208	-1175.1640710	0.5943865	0.0280024	0.0854697
	**9**	-1177.7289802	-1177.6452444	-1177.0919604	-1176.5602454	-1175.1630698	0.5943665	0.0271397	0.0829469
	**10**	-1177.7290367	-1177.6453026	-1177.0918987	-1176.5601913	-1175.1630579	0.5945036	0.0271427	0.0830934
	**11**	-1177.7289484	-1177.6451554	-1177.0918139	-1176.5601409	-1175.1641416	0.5943132	0.0280598	0.0859555
	**12**	-1177.7292901	-1177.6453896	-1177.0921259	-1176.5604412	-1175.1632233	0.5940866	0.0281891	0.0861355
	**13**	-1177.7231058	-1177.6395260	-1177.0873493	-1176.5554829	-1175.1592913	0.5937822	0.0277840	0.0844258
**•C_{10}(DAPANO)$_2^+$**		-1178.1944740	-1178.0799553	-1177.5229670	-1176.9941239	-1175.5945112	0.6070474	0.0307992	0.0895352
**C_{10}PDA**		-851.1872939	-851.1404485	-850.7231146	-850.3410494	-849.3237147	0.4500565	0.0221474	0.0717135
**•C_{10}PDA**	**1**	-850.5175405	-850.4676002	-850.0487633	-849.6703694	-848.6480774	0.4363895	0.0221991	0.0723454
	**1'**	-850.5171468	-850.4672404	-850.0484001	-849.6699488	-848.6484557	0.4363654	0.0221975	0.0723325
	**2**	-850.5226098	-850.4717268	-850.0537405	-849.6743997	-848.6526641	0.4366527	0.0230698	0.0748118
	**3**	-850.5238781	-850.4736022	-850.0558874	-849.6760238	-848.6538204	0.4350047	0.0224913	0.0732458
	**4**	-850.5361067	-850.4860290	-850.0669982	-849.6877706	-848.6654736	0.4367742	0.0220815	0.0724845
	**0**	-850.5055364	-850.4605908	-850.0451334	-849.6572837	-848.6429123	0.4356308	0.0230818	0.0752352
	**5**	-850.5316073	-850.4744372	-850.0646973	-849.6842936	-848.6627480	0.4370821	0.0229817	0.0746583
	**6**	-850.5222623	-850.4748827	-850.0573686	-849.6773197	-848.6560975	0.4354404	0.0224501	0.0730195
	**7**	-850.5228695	-850.4747829	-850.0579764	-849.6778659	-848.6552649	0.4354982	0.0224829	0.0732855
	**8**	-850.5227245	-850.4750252	-850.0578096	-849.6776880	-848.6554238	0.4354703	0.0225293	0.0734720
	**9**	-850.5229053	-850.4562176	-850.0581729	-849.6780025	-848.6367206	0.4354886	0.0234623	0.0767508
	**10**	-850.5229290	-850.4750548	-850.0582195	-849.6780508	-848.6554018	0.4354296	0.0225485	0.0733284
	**11**	-850.5228943	-850.4750341	-850.0581610	-849.6780496	-848.6554455	0.4354377	0.0225657	0.0733143
	**12**	-850.5232337	-850.4752659	-850.0584727	-849.6783477	-848.6565026	0.4352731	0.0226560	0.0734867
	**13**	-850.5168371	-850.4691529	-850.0535627	-849.6732182	-848.6524282	0.4357228	0.0226017	0.0733402
**•C_{10}PDA$^+$**		-850.9655535	-850.9111990	-850.4903835	-850.1120322	-849.0854771	0.4486158	0.0226871	0.0740626
**phenol**		-307.6189992	-307.6027067	-307.4664158	-307.3368484	-306.9399477	0.1011015	0.0057237	0.0358003
**phenol•**		-306.9738902	-306.9582948	-306.8163531	-306.6908902	-306.2889630	0.0887429	0.0054745	0.0361121
**DPPH•**		-1418.6441224	-1418.4479691	-1417.8802788	-1417.3771153	-1415.5825053	0.2786944	0.0242914	0.0773988
**DPPH-H**		-1419.2760072	-1419.0797030	-1418.5116385	-1418.0053798	-1416.2119745	0.2911241	0.0242367	0.0763551
**DPPH$^-$**		-1418.8159720	-1418.6174190	-1418.0537221	-1417.5455846	-1415.7439534	0.2778400	0.0241326	0.0761414
**•OH**		-75.7736546	-75.7756673	-75.7396096	-75.7238826	-75.6434155	0.0081207	0.0023605	0.0202550
**H_2O**		-76.4763367	-76.4744579	-76.4375391	-76.4190458	-76.3415606	0.0204396	0.0028365	0.0221169
**OH^-**		-75.9605418	-75.9575736	-75.9226150	-75.9024033	-75.8049682	0.0083272	0.0023605	0.0195735
**H•**		-0.5000401	-0.5023090	-0.4983070	-0.4987551	-0.4999351	–	–	–

ZPE Zero point energy
TTC Total thermal correction (vibrational, rotational and translational correction)

T 298.15 K
p 1 atm
k_B × T [a.u.] 0.0009442
